# Using External Information for More Precise Inferences in General Regression Models

**DOI:** 10.1007/s11336-024-09953-w

**Published:** 2024-02-20

**Authors:** Martin Jann, Martin Spiess

**Affiliations:** 1 Department of Psychology, University of Hamburg, Von-Melle-Park 5, 20146 Hamburg, Germany; 2 University of Hamburg, Von-Melle-Park 5, 20146 Hamburg, Germany

**Keywords:** external information, generalized method of moments, imprecise probabilities

## Abstract

Empirical research usually takes place in a space of available external information, like results from single studies, meta-analyses, official statistics or subjective (expert) knowledge. The available information ranges from simple means and proportions to known relations between a multitude of variables or estimated distributions. In psychological research, external information derived from the named sources may be used to build a theory and derive hypotheses. In addition, techniques do exist that use external information in the estimation process, for example prior distributions in Bayesian statistics. In this paper, we discuss the benefits of adopting generalized method of moments with external moments, as another example for such a technique. Analytical formulas for estimators and their variances in the multiple linear regression case are derived. An R function that implements these formulas is provided in the supplementary material for general applied use. The effects of various practically relevant moments are analyzed and tested in a simulation study. A new approach to robustify the estimators against misspecification of the external moments based on the concept of imprecise probabilities is introduced. Finally, the resulting externally informed model is applied to a dataset to investigate the predictability of the premorbid intelligence quotient based on lexical tasks, leading to a reduction of variances and thus to narrower confidence intervals.

**Supplementary Information:**

The online version contains supplementary material available at 10.1007/s11336-024-09953-w.

## Introduction

When planning new empirical studies, researchers are confronted with a variety of information from previous studies, including statistical quantities such as means, variances or confidence intervals. However, this external information is mostly used qualitatively, i.e., to develop new theories, and rarely in a quantitative way, i.e., to estimate parameters. One advantage of using external information to estimate a parameter is that some parameter values can be excluded or considered less likely than without the external information, potentially leading to more efficient estimators. The usage of informed prior distributions, where the external information can be used to specify (certain aspects of) the prior distribution, is well known in Bayesian statistics (Bernardo & Smith, 1994). The underlying goal for its use must be clear. On the one hand, external information can facilitate the fitting or tuning of a model. On the other hand, it can make estimators more robust or efficient. This paper aims to achieve the latter of the two goals. Bayesian statistics refers to this as statistical elicitation (Kadane & Wolfson, 1998). The objective is to translate expert knowledge into a prior distribution. Therefore, many psychological biases, such as judgment by representativeness, availability, anchoring, adaptation, or hindsight bias and the intentional misleading by experts, must be considered. It should be noted that the aim is not to achieve objectivity but to ensure a proper statistical representation of subjective knowledge (Garthwaite et al., 2005; Lele & Das, 2000). However, we believe that in applied psychological research, the researcher is usually the one who selects the external information, but is susceptible to the same psychological biases, e.g., in deciding which studies to include. Moreover, the difficulties in eliciting a (multivariate) prior distribution are well documented (Garthwaite et al., 2005, pp. 686–688). The method proposed in this paper allows a simplification of the elicitation compared to Bayesian statistics, since only moments need to be elicited. The elicitation of moments has been well studied for correlations, means, medians, or variances (Garthwaite et al., 2005). In Bayesian elicitation, there are several possible prior distributions for these externally given moments, e.g., with the same expected value or the same correlation, leading to different posterior distributions and thus potentially different results. This problem of prior sensitivity was addressed by Berger (1990) and led to work on robust Bayesian analysis (for an overview, see Insua & Ruggeri, 2000). However, it is somewhat arbitrary to choose the class of distributions for which one wants to make the analysis robust (Garthwaite et al., 2005, p. 695). In our framework, no restriction to a particular class of distributions is required, since it relies solely on moment information and a central limit theorem.

Another important point is that external information may not in general be precise and correct. As nearly all of the external quantities are estimates themselves, they are at least prone to sampling variation. If the external information is not correct (e.g., due to poor sampling or measurement protocols), its use can lead to biased conclusions that may even be worse than without external information. To address this problem, we suggest using an interval for the external information instead of point values, enabling researchers to incorporate any uncertainty about the external moments into the analysis. Inserting external intervals into estimators results in the imprecise probabilistic concept of *feasible probability (F-probability)* discussed in Sect. 4 (Augustin et al., 2014; Weichselberger, 2001). This approach provides an alternative way to enhance the robustness of elicitation compared to the classical Bayesian paradigm: Using intervals can reflect uncertainty about moments, and the resulting inference is still coherent if the interval contains the true value. However, researchers must be cautious of and avoid overconfidence bias when eliciting intervals; that is, the tendency to select intervals that are too narrow to represent current uncertainty (Winman et al., 2004). A test of the latter assumption is available, more specifically a test of the compatibility of the external interval and the data, which could serve as a pretest before applying the methods proposed here (Jann, 2023).

The insertion of intervals into estimators resembles creating fuzzy numbers (Kwakernaak, 1978; Zadeh, 1965), for which generalizations of traditional statistical methods already exist. This is particularly true for the special case of triangular numbers (Buckley, 2004). The possibility distributions induced by triangular numbers constitute special cases of imprecise probabilities and are constructed based on only one distribution (Augustin et al., 2014, pp. 84–87). This is the key difference between triangular numbers and F-probabilities, since the latter are constructed from a set of possible probability distributions, which can enhance the robustness of the outcomes compared to constructions based on only one distribution. Another difference lies in the fact that triangular numbers are constructed by varying the confidence probability of a confidence interval based on the estimator, while the external interval we use in this paper is fixed. Moreover, there is no probabilistic statement about the values within that interval.

In the present study, we analyze the frequentist properties of estimators if external information is used, that can be expressed as moment conditions and thus does not use complete distributions as prior information. To our knowledge, there is no general framework for robustly incorporating such quantitative external information into frequentist analysis. Since this would offer the advantage of improving upon classical inference procedures widely used in psychology, our goal is to present such a framework. The use of these external moment conditions in addition to the moment conditions used to estimate the model parameters leads to an overidentified system of moment conditions. The main idea to find well performing estimators for such “externally” overidentified systems is the framework of the Generalized Method of Moments (GMM) (Hansen, 1982). This idea has already been used in the econometric literature, for example, by Imbens and Lancaster (1994) who combine micro- and macro-economic data and by Hellerstein and Imbens (1999) by constructing weights for regression models based on auxiliary data. A different yet related way to incorporate external moment-information is the empirical likelihood approach (Owen, 1988). This technique is quite frequently used in the literature, for example, in finite population estimation (Zhong & Rao, 2000) and for externally informed generalized linear models (Chaudhuri et al., 2008). Both approaches have in common that the use of external information may increase the efficiency of an estimator and/or reduce its bias.

Actually, in Sect. 3, we show that there will always be a variance reduction, if the external moment conditions and the ones for the model are correlated and if the covariance matrix of all moment conditions is positive definite. As the GMM allows the estimation of a large class of models, and many statistical measures like proportions, means, variances and covariances are statistical moments, the range of possible applications is large but far from being implemented in psychological research. For a multiple linear model, we derive the estimators analytically in Sect. 3. The use of imprecise probabilities will increase the overall variation of the estimator, and moreover, the effect of the variance reduction will decrease. As we will demonstrate, however, variance reduction will still be possible while increasing the robustness of the estimation. The proposed method and techniques allow more precise and robust inferences, which is particularly relevant in small samples. To illustrate the small sample performance of the externally informed models in multiple linear models, a simulation study is presented in Sect. 5. An application to a real data set analyzing the relation of premorbid (general) intelligence and performance in lexical tasks (Pluck & Ruales-Chieruzzi, 2021) is presented in Sect. 6.

## Externally Informed Models

In a first step, we assume that precise external information is available, an assumption that will be relaxed in Sect. 4. Throughout, we assume that all variables will be considered as random variables if not given otherwise. For notational clarity, we will always write single-valued random variables in italic small letters. Vectors as well as vector-valued functions will be written in small bold letters and matrices in bold capital letters.

Although the basic concepts are presented in the following section, for the class of general regression models, we will consider the family of linear models for their illustration in a concrete class of models due to their frequent use. Note that, for example, ANOVA models are special cases of this model, however, with fixed factors instead of random covariates. Nevertheless, the results derived in this paper carry over to these models.

Let ${{\textbf {z}}}=(z_1,\dots ,z_p)^T$ be a real-valued random vector and ${{\textbf {z}}}_i$, $i=1,\dots , n$, be i.i.d. random vectors distributed like ${{\textbf {z}}}$, representing the data. Suppose we want to fit a regression model to this data set with fixed parameter $\varvec{\theta } \in \mathbb {R}^p$, where the adopted model reflects the interesting aspects of the true data-generating process and $\varvec{\theta }_0$ is the true parameter value. In linear regression models, the parameter of scientific interest is usually the parameter of the mean structure denoted as $\varvec{\beta }=(\beta _1,\dots ,\beta _{p})^T$ with true value $\varvec{\beta }_0$. The notation $\varvec{\beta }$ will only be used for linear regression models, while we will use $\varvec{\theta }$ to denote the regression coefficients in general regression models. The random vector **z** is given by ${{\textbf {z}}}=({{\textbf {x}}}^T,y)^T$ with random explanatory variables ${{\textbf {x}}}=(x_1,\dots ,x_{p})^T$ and dependent variable *y*. Accordingly, the unit specific i.i.d. random vectors **z** are written as ${{\textbf {z}}}_i=({{\textbf {x}}}_i^T,y_i)^T$ for $i=1,\dots , n$. Hence, the random $(n \times p)$-design matrix is ${{\textbf {X}}}=({{\textbf {x}}}_1,\dots ,{{\textbf {x}}}_n)^T$, and we write ${{\textbf {y}}}=(y_1,\dots ,y_n)^T$.

The multiple linear model can now be written as ${{\textbf {y}}}={{\textbf {X}}}\varvec{\beta }_0 + \varvec{\epsilon }$ with random error terms $\varvec{\epsilon }=(\epsilon _1,\dots ,\epsilon _n)^T$. As an illustration, suppose we want to investigate the effect of the explanatory variables *fluid intelligence* and *depression* on the dependent variable *mathematics skills*. We could design a study, in which fluid intelligence and math skills are measured via Cattell’s fluid intelligence test, in short CFT 20-R, ($x_2$) and the number sequence test ZF-R (*y*), respectively (Weiss, 2006). Depression could be measured as a binary variable indicating if a person has a depression-related diagnosis ($x_3$). The model could be a linear multiple regression of the ZF-R score on the depression indicator and the CFT 20-R score for fluid intelligence. To include the intercept, $x_1$ is a degenerate variable with value 1.

In addition to the observed data and the assumptions justifying the model, we often have available external information like means, correlations or proportions, e.g., through official statistics, meta-analyses or already existing individual studies. In our applied example, there are various German norm groups for the CFT 20-R and the ZF-R, even for different ages (Weiss, 2006). Hence, we could always transform the results into scores with known expected value and variance, i.e. the CFT 20-R score can be transformed into an IQ-score based on a recent calibration sample from 2019, reported in the test manual (Weiss, 2019). Regarding the relation of fluid intelligence and math skills, a recent meta-analysis based on more than 370,000 participants in 680 studies from multiple countries suggests a correlation of $r=0.41$ between the two variables (Peng et al., 2019). In addition, based on a study covering 87$\%$ of the German population aged at least 15 years, Steffen et al. (2020) report a prevalence of depression, defined as a F32, F33 or F34.1 diagnosis following the ICD-10-GM manual, of $15.7\%$ in 2017.

Let us assume that these values can be interpreted as true population values, an assumption that will be relaxed later. Note that they have the form of statistical moments. For example, the observable depression prevalence is assumed to equal the expected value of the binary depression indicator (first moment), the mean (now considered as expected value) and variance of the test scores are set equal to the first moment and the second central moment, respectively, of the random variables CFT 20-R-score and ZF-R-score. Finally, the correlation is assumed to equal the mixed moment of the standardized CFT 20-R-score and ZF-R-score. Taking *q* to be the number of known external moments, we state

### Definition 1

Let *M* be a statistical model. Further let ${{\textbf {u}}}$ be a $(q \times 1)$-vector of statistical moment expressions and $\varvec{\mu }_{\textrm{ex}}$ the corresponding $(q \times 1)$-vector of externally determined values for the statistical moments in ${{\textbf {u}}}$. Then the model combining *M* and the conditions ${{\textbf {u}}} = \varvec{\mu }_{\textrm{ex}}$ is called *externally informed model*.

To illustrate the definition, we will use the applied example from above in which case the model *M* is a multiple linear regression model. Interpreting the norms for the dependent variable ZF-R from the calibration sample as population values, external knowledge about the corresponding moments, for example the means of ZF-R, is available. Let us assume that ZF-R is transformed into the IQ-scale. Then, if ${{\textbf {u}}} = E(y)$ and $\varvec{\mu }_{\textrm{ex}}=100$, we get $E({{\textbf {y}}}) = 100 \times {\varvec{1}}_n = E({{\textbf {X}}})\varvec{\beta }_0$, where ${\varvec{1}}_n$ is a $(n \times 1)$-vector of ones. Thus, ${{\textbf {u}}}=\varvec{\mu }_{\textrm{ex}}$ imposes conditions on $\varvec{\beta }$.

## Estimation and Properties of Externally Informed Models

### Generalized Method of Moments with External Moments

The GMM approach (Hansen, 1982) allows to estimate (general) regression models and to incorporate external moments into the estimation (Imbens & Lancaster, 1994). To estimate the parameter of a general regression model, a “model moment function” ${{\textbf {m}}}({{\textbf {z}}},\varvec{\theta })$ must be given, which satisfies the conditions $E[{{\textbf {m}}}({{\textbf {z}}},\varvec{\theta })] = {{\textbf {0}}}$ only for the true parameter value $\varvec{\theta }_0$. The corresponding “sample moment function” for ${{\textbf {z}}}_i$ will be denoted as ${{\textbf {m}}}({{\textbf {z}}}_i,\varvec{\theta })$. In case of the linear regression model from Sect. 2, the model moment function corresponding to the method of Ordinary Least Squares (OLS) is ${{\textbf {m}}}({{\textbf {z}}},\varvec{\beta }) = {{\textbf {x}}}(y-{{\textbf {x}}}^T\varvec{\beta })$ (Cameron & Trivedi, 2005, p. 172). Given the model is correctly specified, for true parameter value $\varvec{\beta }_0$, $E[{{\textbf {m}}}({{\textbf {z}}},\varvec{\beta }_0)] = E[{{\textbf {x}}}(y-{{\textbf {x}}}^T\varvec{\beta }_0)] = {{\textbf {0}}}$ holds. Replacing these population model moment conditions by corresponding sample model moment conditions,

$$\begin{aligned} {{\textbf {0}}}= \frac{1}{n}\sum _{i=1}^n {{\textbf {m}}}({{\textbf {z}}}_i,\varvec{\beta }) = \frac{1}{n}\sum _{i=1}^n {{\textbf {x}}}_i\left( y_i-{{\textbf {x}}}_i^T\varvec{\beta }\right) =\frac{1}{n}{{\textbf {X}}}^T({{\textbf {y}}}-{{\textbf {X}}}\varvec{\beta }) \; , \end{aligned}$$

and solving these estimating equations for $\varvec{\beta }$, leads to an estimator $\hat{\varvec{\beta }}$ for $\varvec{\beta }_0$. The above conditions are identical to the estimating equations resulting from the least-squares or, if normality of the errors is assumed, the maximum likelihood method. Furthermore, the general classes of *M*- and *Z*-estimators can be written using estimating equations that have this moment form. This leads to broad applicability, since these classes, for example, include the median and quantiles (Vaart, 1998).

The possibly vector-valued “external moment function” will be denoted as ${{\textbf {h}}}({{\textbf {z}}}) = {{\textbf {u}}}({{\textbf {z}}}) - \varvec{\mu }_{\textrm{ex}}$, where the functional form of ${{\textbf {u}}}({{\textbf {z}}})$ depends on the external information included into the model. We assume that $\varvec{\mu }_{\textrm{ex}} = E[{{\textbf {u}}}({{\textbf {z}}})]$, so that $E[{{\textbf {h}}}({{\textbf {z}}})] = {\varvec{0}}$. If, for example, the expected value of *y* is known to be $E(y) = 100$, then $u(z) = y$, $\mu _{\textrm{ex}} = 100$ and $h(z) = y - 100$. The corresponding sample moment condition is $0 = \frac{1}{n} \sum _{i=1}^n (y_i - 100)$ (Imbens & Lancaster, 1994).

To simplify the presentation, we define the combined moment function vector in general regression models as ${{\textbf {g}}}({{\textbf {z}}},\varvec{\theta })=[{{\textbf {m}}}({{\textbf {z}}},\varvec{\theta })^T,{{\textbf {h}}}({{\textbf {z}}})^T]^T$ in what follows and assume that $E[\frac{1}{n} \sum _{i=1}^n {{\textbf {g}}}({{\textbf {z}}}_i,\varvec{\theta }_0)] = {\varvec{0}}$ holds. Note that the number of moment conditions exceeds the number of parameters to be estimated, i.e. the externally informed model is overidentified. This means that there will in general be no estimator $\hat{\varvec{\theta }}$ that solves the corresponding sample moment conditions $\frac{1}{n} \sum _{i=1}^n {{\textbf {g}}}({{\textbf {z}}}_i,\varvec{\theta }) = {\varvec{0}}$. To deal with the overidentification problem, we will use the GMM approach (Hansen, 1982), that finds an estimator as “close” as possible to a solution of the sample moment conditions. This is done by maximizing a quadratic form defined by a chosen symmetric, positive definite weighting matrix **W** in the moment functions of the sample. The efficiency of the estimator is affected by **W**, and this can be chosen to maximize the asymptotic efficiency of the estimator in the class of all GMM-estimators based on the same sample moment conditions (Hansen, 1982). This optimal weighting matrix is ${{\textbf {W}}}=\varvec{\Omega }^{-1}$, where $\varvec{\Omega }={E}[{{\textbf {g}}}({{\textbf {z}}},\varvec{\theta }_0){{\textbf {g}}}({{\textbf {z}}},\varvec{\theta }_0)^T]$. However, this optimal **W** is unknown in practice and must be estimated by a consistent estimator $\hat{{{\textbf {W}}}}$.

#### Definition 2

(Newey & McFadden, 1994, p. 2116) Let ${{\textbf {g}}}({{\textbf {z}}},\varvec{\theta })$ be a vector-valued function with values in $\mathbb {R}^K$, that meets the moment conditions $E[{{\textbf {g}}}({{\textbf {z}}},\varvec{\theta }_0)] = {{\textbf {0}}}$. Further let $\hat{{{\textbf {W}}}} \in \mathbb {R}^{K,K}$ be a positive-semidefinite, possibly random matrix, such that $({{\textbf {r}}}^T \hat{{{\textbf {W}}}}{{\textbf {r}}})^{1/2}$ is a measure of distance from ${{\textbf {r}}}$ to ${{\textbf {0}}}$ for all ${{\textbf {r}}} \in \mathbb {R}^K$. Then, the **GMM-estimator** $\hat{\varvec{\theta }}_{\textrm{ex}}$ is defined as the $\varvec{\theta }$, which maximizes the following function:

$$\begin{aligned} {\hat{Q}}_n(\varvec{\theta })= - \left[ \frac{1}{n} \sum _{i=1}^n {{\textbf {g}}}({{\textbf {z}}}_i,\varvec{\theta })\right] ^T \hat{{{\textbf {W}}}} \left[ \frac{1}{n} \sum _{i=1}^n{{\textbf {g}}}({{\textbf {z}}}_i,\varvec{\theta })\right] \; . \end{aligned}$$

The GMM approach provides consistent and normally distributed estimators under mild regularity conditions (Newey & McFadden, 1994, p. 2148) for a wide range of models, like linear or nonlinear, cross-sectional or longitudinal regression models. Note that we have not assumed that $\hat{{{\textbf {W}}}}$ is invertible because we will mainly derive asymptotic expressions based on **W** for which the invertibility of $\hat{{{\textbf {W}}}}$ is not necessary. However, when deriving estimators, additional assumptions about invertibility must be made, which we explain in Sect. 3.2. Let ${{\textbf {G}}}={E}[\nabla _{\varvec{\theta }} {{\textbf {g}}}({{\textbf {z}}},\varvec{\theta }_0)]$ be a fixed matrix and **W** the optimal weighting matrix, then $\text {Var}(\hat{\varvec{\theta }}_{\textrm{ex}}) = \frac{1}{n} ({{\textbf {G}}}^T {{\textbf {W}}}{\varvec{G}})^{-1}$. This variance expression is not informative with respect to a possible efficiency gain of the GMM-estimator if external information is used. Hence, the following corollary explicitly shows the effect of the external information on the variance of $\hat{\varvec{\theta }}_{\textrm{ex}}$.

#### Corollary 1

Assume $\hat{\varvec{\theta }}_M$ is the GMM-estimator based on the model estimating equations alone (ignoring the external moments), and that ${{\textbf {m}}}({{\textbf {z}}},\varvec{\theta })$ and $\varvec{\theta }$ have the same dimension. Using the prerequisite ${{\textbf {g}}}({{\textbf {z}}},\varvec{\theta })=[{{\textbf {m}}}({{\textbf {z}}},\varvec{\theta })^T,{{\textbf {h}}}({{\textbf {z}}})^T]^T$ it follows, that $\varvec{\Omega }$ has the block form

$$\begin{aligned} \varvec{\Omega }= \left( \begin{array}{cc} E[{{\textbf {m}}}({{\textbf {z}}},\varvec{\theta }){{\textbf {m}}}({{\textbf {z}}},\varvec{\theta })^T] &{}\quad E[{{\textbf {m}}}({{\textbf {z}}},\varvec{\theta }){{\textbf {h}}}({{\textbf {z}}})^T ] \\ E[{{\textbf {h}}}({{\textbf {z}}}){{\textbf {m}}}({{\textbf {z}}},\varvec{\theta })^T] &{}\quad E[{{\textbf {h}}}({{\textbf {z}}}){{\textbf {h}}}({{\textbf {z}}})^T] \end{array}\right) = \left( \begin{array}{cc} \varvec{\Omega }_{M} &{}\quad \varvec{\Omega }_R^T \\ \varvec{\Omega }_R &{}\quad \varvec{\Omega }_h \end{array}\right) \end{aligned}$$

and that

1 $$\begin{aligned} \left\{ E[\nabla _{\varvec{\theta }} {{{\textbf {m}}}}({{{\textbf {z}}}},\varvec{\theta }_0)]^T\right\} ^{-1} \varvec{\Omega }_{R}^T\varvec{\Omega }_{h}^{-1}\varvec{\Omega }_{R} \left\{ E[\nabla _{\varvec{\theta }} {{{\textbf {m}}}}({{{\textbf {z}}}},\varvec{\theta }_0)]\right\} ^{-1} \end{aligned}$$

A proof of Corollary 1 can be found in the supplementary materials online. Note that (1) shows that $\text {Var}(\hat{\varvec{\theta }}_{\textrm{ex}})$ is equal to the conditional variance of $\hat{\varvec{\theta }}_{M}$ under the external moment conditions, since the asymptotic distribution is normal. This equality shows why there is a reduction in the variance. Let the second term on the right-hand side of (1) be denoted by **D**, then ${\textrm{Var}}(\hat{\varvec{\theta }}_{\textrm{ex}})$ can be written as ${\textrm{Var}}(\hat{\varvec{\theta }}_{\textrm{ex}}) = {\textrm{Var}}(\hat{\varvec{\theta }}_M) - {{\textbf {D}}}$. If ${{\textbf {D}}}$ is nonnegative definite and not equal to ${{\textbf {0}}}$, then including external moments leads to an expected efficiency gain in $\hat{\varvec{\theta }}_{\textrm{ex}}$ as compared to $\hat{\varvec{\theta }}_M$. That ${{\textbf {D}}}\ne {{\textbf {0}}}$ is nonnegative definite if $\varvec{\Omega }_R \ne {{\textbf {0}}}$ is easily seen by noting that $\varvec{\Omega }_h^{-1}$ is positive definite and therefore can be written as $\varvec{\Omega }_h^{-1} =\varvec{\Omega }_h^{-1/2}\varvec{\Omega }_h^{-1/2}$, where $\varvec{\Omega }_h^{-1/2}$ is the positive definite square root of $\varvec{\Omega }_h^{-1}$. Since $n {{\textbf {D}}}$ can be written as the product of $\{E[\nabla _{\varvec{\theta }} {{\textbf {m}}}({{\textbf {z}}},\varvec{\theta }_0)]^T\}^{-1} \varvec{\Omega }_{R}^T\varvec{\Omega }_{h}^{-1/2}$ with its transpose, ${{\textbf {D}}}$ is nonnegative definite. In summary, $\varvec{\Omega }_R \ne {{\textbf {0}}}$ is a necessary and sufficient condition for the presence of variance reduction based on Corollary 1. Finally, it should be noted that $\text {Var}(\hat{\varvec{\theta }}_{\textrm{ex}})$ can consistently be estimated via the plug-in approach (e.g. Newey & McFadden, 1994, pp. 2171–2173) by replacing all unknown expected values by sample means.

### The Externally Informed Multiple Linear Model

In linear models, $\hat{\varvec{\theta }}_{\textrm{ex}}$ is denoted as $\hat{\varvec{\beta }}_{\textrm{ex}}$. For analytical simplicity, in this section, we assume the Gauss–Markov assumptions hold, specifically $E(\epsilon _i)=0, Var(\epsilon _i)=\sigma ^2, Cov( \epsilon _i,\epsilon _j)=0$ for all $i\ne j$ with $i,j = 1, \dots , n$, and independence of the explanatory variables and the error terms $\varvec{ \epsilon }$. Furthermore, we assume the errors to be normally distributed in small samples. Analytical solutions to the estimating equations exist under these assumptions:

#### Theorem 1

Let ${{\textbf {H}}}=[ {{\textbf {h}}}({{\textbf {x}}}_1,y_1),\dots ,{{\textbf {h}}}({{\textbf {x}}}_n,y_n)]^T$ be the $(n \times q)$ random matrix containing the externally informed sample moment functions and ${{\textbf {1}}}_n$ a $(n \times 1)$-vector of ones. Further let $\hat{\varvec{\Omega }}_h$ and $\hat{\varvec{\Omega }}_R$ be consistent estimators of the corresponding matrices in Corollary 1. Then, the (consistent) externally informed OLS estimator is:

$$\begin{aligned} \hat{\varvec{\beta }}_{\textrm{ex}}= ({{\textbf {X}}}^T{{\textbf {X}}})^{-1}{{\textbf {X}}}^T{{\textbf {y}}}-({{\textbf {X}}}^T{{\textbf {X}}})^{-1}\hat{\varvec{\Omega }}_R^T \hat{\varvec{\Omega }}_h^{-1}{{\textbf {H}}}^T{{\textbf {1}}}_n \end{aligned}$$

and its variance is

$$\begin{aligned} {\textrm{Var}}(\hat{\varvec{\beta }}_{\textrm{ex}})&= {\textrm{Var}}(\hat{\varvec{\beta }}) - {{\textbf {D}}} \\ {}&= \frac{1}{n}\sigma ^2\left[ E\left( {{\textbf {x}}}{{\textbf {x}}}^T\right) \right] ^{-1}-\frac{1}{n}\left[ E\left( {{\textbf {x}}}{{\textbf {x}}}^T\right) \right] ^{-1}\varvec{\Omega }_R^T \varvec{\Omega }_h^{-1}\varvec{\Omega }_R \left[ E\left( {{\textbf {x}}}{{\textbf {x}}}^T\right) \right] ^{-1}, \end{aligned}$$

where $\sigma ^2$ is the variance of the error in the assumed linear model.

The proof of Theorem 1 is given in the supplementary materials online. Note that only the assumption of invertibility of $\hat{\varvec{\Omega }}_h$ is made, which is weaker than the assumption that $\hat{\varvec{\Omega }}$ is invertible. From Theorem 1, it is not immediately obvious which of several possibly available functions may lead to a variance reduction. Therefore, let us consider some external moment functions and their possible effects on the variance of $\hat{\varvec{\beta }}_{\textrm{ex}}$. Note that inclusion of external moment functions into the estimating equations may lead to expected efficiency gains only if $\varvec{\Omega }_R^T = E[{{\textbf {x}}} (y - {{\textbf {x}}}^T \varvec{\beta }_0) \text {{\textbf {h}}}(\text {{\textbf {x}}}, y)^T] = E[{{\textbf {x}}} \, \epsilon \, \text {{\textbf {h}}}(\text {{\textbf {x}}}, y)^T] \ne {{\textbf {0}}}$ holds.

Let the expressions $\sigma _{x_j}$ and $\sigma _y$ denote the population standard deviations of $x_j$ and *y*, respectively, whereas $\sigma _{x_j,y}$ indicates the covariance of $x_j$ and *y*. To denote the covariance vector $(\sigma _{x_1,x_j}, \dots , \sigma _{x_p,x_j})^T$ of ${{\textbf {x}}}$ and $x_j$ the expression $\varvec{\sigma }_{x_{\cdot },x_j}$ is used, including $\sigma ^2_{x_j}$ at the *j*-th position. Finally, $\rho _{x_j,y}$ is the population correlation of $x_j$ and *y*.

First, consider some function ${{\textbf {f}}}({{\textbf {x}}})$ of ${{\textbf {x}}}$, i.e. $\text {{\textbf {h}}}({{\textbf {x}}}) = {{\textbf {f}}}({{\textbf {x}}}) - E[{{\textbf {f}}}({{\textbf {x}}})]_{\textrm{ex}}$, where $E[{{\textbf {f}}}({{\textbf {x}}})]_{\textrm{ex}}$ is the known expected value of ${\textbf {f(x)}}$. If the assumptions underlying the linear model hold, then $\varvec{\Omega }_R=E[{{\textbf {x}}} \, \epsilon \, \text {{\textbf {h}}}(\text {{\textbf {x}}})^T] = {{\textbf {0}}}$ because $\epsilon$ is independent of ${\textbf {f(x)}}$ and $E(\epsilon ) = 0$. Thus, according to the results of Sect. 3.1, there will be no variance reduction if the external moment function is a function of the explanatory variables only. In the example described in Sect. 2, there will be no efficiency gain if the $15.7\%$-prevalence of depression is used as external information to estimate the linear regression model.

On the other hand, if the external moment function is a function of $\epsilon$, then generally, $E[{{\textbf {x}}} \, \epsilon \, \text {{\textbf {h}}}(\text {{\textbf {x}}}, y)^T] \ne {{\textbf {0}}}$. In the example, assume that the correlation between fluid intelligence and math skills reported in Peng et al. (2019) is taken as external information, in which case $\text {{\textbf {h}}}(\text {{\textbf {x}}}, y)=h(x_2, y) = [y-E(y)][x_2-E(x_2)] /(\sigma _{x_2} \sigma _y) - \rho (x_2, y)_{\textrm{ex}}$, where $\rho (x_2, y)_{\textrm{ex}} = 0.41$. Then $E[{{\textbf {x}}} \, \epsilon \, h(x_2, y)] = [\sigma ^2 /(\sigma _{x_2}\sigma _y)] \varvec{\sigma }_{x_{\cdot },x_2}$ will not in general be zero, and hence, there will, in general, be efficiency gains with respect to $\hat{\varvec{\beta }}_{\textrm{ex}}$. For more examples, see Table 1 and for the derivation of the results, see the supplementary materials online. It should be noted that if the distribution of the errors is not symmetrical, then $E({{\textbf {x}}})E(\epsilon ^3)$ has to be added to the entries in column $\varvec{ \Omega }_R^T$ of Table 1 for the cases $E(y^2)$ and $\sigma _y^2$, see the supplementary materials online for further details.

**Table 1:** Forms of $\varvec{ \Omega }_R^T$ for various single moments.

Moments	$h({{\textbf {x}}},y)$	$\varvec{ \Omega }_R^T$
*E*(*y*)	$y-E(y)_{\textrm{ex}}$	$\sigma ^2 E({{\textbf {x}}})$
$E(x_jy)$	$x_jy- E(x_jy)_{\textrm{ex}}$	$\sigma ^2 E(x_j\cdot {{\textbf {x}}})$
$E(y^2)$	$y^2-E(y^2)_{\textrm{ex}}$	$2 \sigma ^2 E({{\textbf {x}}} {{\textbf {x}}}^T) \varvec{\beta }_0$
$\sigma _y^2$	$[y-E(y)]^2-(\sigma _y^2)_{\textrm{ex}}$	$2 \sigma ^2 [E({{\textbf {x}}} {{\textbf {x}}}^T ) \varvec{\beta }_0 - E(y)E({{\textbf {x}}})]$
$\sigma _{x_j,y}$	$[y-E(y)][x_j-E(x_j)]-(\sigma _{x_j,y})_{\textrm{ex}}$	$\sigma ^2 \varvec{\sigma }_{x_{\cdot },x_j}$
$\rho _{x_j,y}$	$\frac{[y-E(y)][x_j-E(x_j)]}{\sigma _{x_j} \sigma _y}-(\rho _{x_j,y})_{\textrm{ex}}$	$\frac{\sigma ^2}{\sigma _{x_j}\sigma _y} \varvec{\sigma }_{x_{\cdot },x_j}$
$\beta _{x_j,y}$	$\frac{[y-E(y)][x_j-E(x_j)]}{\sigma _{x_j}^2}-(\beta _{x_j,y})_{\textrm{ex}}$	$\frac{\sigma ^2}{\sigma _{x_j}^2} \varvec{\sigma }_{x_{\cdot },x_j}$

The subscript ex indicates externally determined values. In the last line, $\beta _{x_j,y}$ represents the expected value of the estimator of the slope from a simple linear regression model, which is identical to the true value of the slope only if $x_j$ is independent of the other explanatory variables.

**Table 2:** Effects of various single moments in terms of variance reduction.

Moments	${{\textbf {D}}}$	Effect on
*E*(*y*)	$\frac{\sigma ^4}{n\omega _h}{{\textbf {e}}}_1{{\textbf {e}}}_1^T$	Only $\beta _1$
$E(x_jy)$	$\frac{\sigma ^4}{n\omega _h}{{\textbf {e}}}_j{{\textbf {e}}}_j^T$	Only $\beta _{j}$
$E(y^2)$	$\frac{4\sigma ^4}{n\omega _h}\varvec{\beta }_0\varvec{\beta }_0^T$	All $\beta _j \ne 0$
$\sigma _y^2$	$\frac{4\sigma ^4}{n\omega _h}[\varvec{\beta }_0-E(y){{\textbf {e}}}_1][\varvec{\beta }_0-E(y){{\textbf {e}}}_1]^T$	All $\beta _j \ne 0$ and $\beta _1$
$\sigma _{x_j,y}$	$\frac{\sigma ^4}{n\omega _h}\tilde{{{\textbf {e}}}}_j\tilde{{{\textbf {e}}}}_j^T$	$\beta _{j}$ and $\beta _1$
$\rho _{x_j,y}$	$\frac{\sigma ^4}{n\omega _h\sigma _y^2\sigma _{x_j}^2}\tilde{{{\textbf {e}}}}_j\tilde{{{\textbf {e}}}}_j^T$	$\beta _{j}$ and $\beta _1$
$\beta _{x_j,y}$	$\frac{\sigma ^4}{n\omega _h\sigma _{x_j}^4}\tilde{{{\textbf {e}}}}_j\tilde{{{\textbf {e}}}}_j^T$	$\beta _{j}$ and $\beta _1$

The expression ${{\textbf {e}}}_j$ denotes the $(p \times 1)$-vector with 1 at the *j*-th position and zeros elsewhere. Further we set $\tilde{{{\textbf {e}}}}_j:= -E(x_j)\cdot {{\textbf {e}}}_1 + {{\textbf {e}}}_j$. In the last line, $\beta _{x_j,y}$ represents the expected value of the estimator of the slope from a simple linear regression model, which is identical to the true value of the slope only if $x_j$ is independent of the other explanatory variables.

Table 2 presents, in the second column, the absolute variance reduction for the parameters if the external information given in the first column is used to estimate the regression model. The third column in Table 2 shows which entries of the parameter $\varvec{\beta }$ can be estimated more precisely if the external information is used. The results of Table 2 are derived in the supplementary materials online. Note that $\varvec{\Omega }_h$ is written as $\omega _h$ here, as it is single-valued. It holds that $\omega _h=E[h({{\textbf {x}}},y)^2]$, where $h({{\textbf {x}}},y)$ is of the form given for various moments in Table 1. However, this expression often includes the terms $E(\epsilon )$ and $E(\epsilon ^3)$, which are already set to zero in $\varvec{ \Omega }_{R}^T$ (see supplementary materials online). In order to avoid invalid estimates, $E(\epsilon )$ and $E(\epsilon ^3)$ should be set to zero in $\omega _{h}$. For example, if the correlation between fluid intelligence and math skills reported in Peng et al. (2019) would be used in the regression from math skills on fluid intelligence and depression, then the variance of the estimator weighting the variable fluid intelligence would be reduced by:

$$\begin{aligned} \frac{\sigma ^4}{n\omega _h\sigma _y^2\sigma _{x_2}^2}=\frac{\sigma ^4}{n \text {Var}\{[x_2-E(x_2)][y-E(y)]\}}. \end{aligned}$$

This means that there will be a variance reduction in all practically relevant cases, where $\sigma ^2\ne 0$ and $\text {Var}\{[x_2-E(x_2)][y-E(y)]\}<\infty$ hold. For a comparison of the effects of the different external moments, the corresponding relative variance reductions may be of interest. These are obtained by dividing the *j*-th diagonal element of the absolute reductions in Table 2 by $\frac{1}{n}\sigma ^2 E({{\textbf {x}}}{{\textbf {x}}}^T)^{-1}_{(j,j)}$, where $E({{\textbf {x}}}{{\textbf {x}}}^T)^{-1}_{(j,j)}$ denotes the element of the inverse of $E({{\textbf {x}}}{{\textbf {x}}}^T)$ in the *j*-th row and the *j*-th column. For the resulting expressions it is clear that *n* factors out, as ${{\textbf {D}}}$ also includes $\frac{1}{n}$ as the only factor depending on *n*, while the rest are fixed values. Hence, the relative efficiency gains do not vanish with increasing *n*, but are constant. In our example, the known correlation $\rho _{x_2,y}=.41$ exerts an expected relative variance reduction of

$$\begin{aligned} \frac{\sigma ^2}{E({{\textbf {x}}}{{\textbf {x}}}^T)^{-1}_{(2,2)}{\text {Var}}\{[x_2-E(x_2)][y-E(y)]\}}, \end{aligned}$$

which is independent of *n* and does not vanish for large $\sigma ^2$. Including more than one external moment is straightforward. In that case $\varvec{\Omega }_h$ includes not only variances but also covariances of the external moments which may lead to additional variance reduction. To illustrate this effect, consider the example from Sect. 2 using the external moments $\rho (x_2, y)_{\textrm{ex}} = 0.41$ and $E(x_2)_{\textrm{ex}} = 100$. For the sake of simplicity and without loss of generality we assume $x_2$ and *y* to be centralized. In this example, the external moments $\rho _{x_2,y}$ and $E(x_2)$ are included in the externally informed multiple linear model, leading to $$\varvec{\Omega }_R^T=\begin{pmatrix} {{\textbf {0}}}&\frac{\sigma ^2}{\sigma _{x_2}\sigma _y} \varvec{\sigma }_{x_{\cdot },x_j} \end{pmatrix}$$ according to Table 1, and

$$\begin{aligned} \varvec{\Omega }_h= \begin{pmatrix} \text {Var}(x_2) &{}\quad \frac{\text {Cov}(x_2^2,y)}{\sigma _{x_2}\sigma _y} \\ \frac{\text {Cov}(x_2^2,y)}{\sigma _{x_2}\sigma _y} &{}\quad \frac{\text {Var}(x_2y)}{\sigma _{x_2}^2\sigma _y^2} \end{pmatrix} \end{aligned}$$

via definition, wherein $\text {Var}(x_2y)$ is the scalar variance of $x_2$ times *y*. Using the notation of Table 2, the explicit inversion formula for ($2 \times 2$)-matrices implies

$$\begin{aligned} {{\textbf {D}}}&=\frac{1}{n}\left[ E\left( {{\textbf {x}}}{{\textbf {x}}}^T\right) \right] ^{-1}\varvec{\Omega }_R^T \varvec{\Omega }_h^{-1}\varvec{\Omega }_R \left[ E\left( {{\textbf {x}}}{{\textbf {x}}}^T\right) \right] ^{-1} \\ {}&= \frac{1}{n}\left[ E\left( {{\textbf {x}}}{{\textbf {x}}}^T\right) \right] ^{-1} \frac{\sigma ^2}{\sigma _{x_2}\sigma _y} \varvec{\sigma }_{x_{\cdot },x_j} (\varvec{ \Omega }_{h}^{-1})_{(2,2)} \varvec{\sigma }_{x_{\cdot },x_j}^T \frac{\sigma ^2}{\sigma _{x_2}\sigma _y} \left[ E\left( {{\textbf {x}}}{{\textbf {x}}}^T\right) \right] ^{-1}\\ {}&= \frac{\sigma ^4(\varvec{ \Omega }_h)_{(1,1)}}{n\det (\varvec{ \Omega }_h)\sigma _{x_2}^2\sigma _y^2} \tilde{{{\textbf {e}}}}_2 \tilde{{{\textbf {e}}}}_2^T = \frac{\sigma ^4}{n\left[ \text {Var}(x_2y)-\frac{\text {Cov}\left( x_2^2,y\right) ^2}{\sigma ^2_{x_2}} \right] }\tilde{{{\textbf {e}}}}_2 \tilde{{{\textbf {e}}}}_2^T, \end{aligned}$$

where $\det ({{\textbf {A}}})$ denotes the determinant of matrix **A**. Assuming both variances to be finite and positive and invoking the Cauchy–Schwartz inequality, the fraction $\text {Cov}(x^2_2,y)^2 / \sigma ^2_{x_2}$ will not exceed $\text {Var}(x_2y)$ and hence ${{\textbf {D}}}$ will be nonnegative. Further, if $x^2_2$ and *y* have a covariance different from 0, the variance will decrease even further, compared to the reduction due to $\rho _{x_2,y}$ alone. Hence, $\beta _1$ and $\beta _2$ can in general be estimated even more efficiently, if $E(x_2)$ is used in addition.

### Additional Remarks

Using many moments, however, increases the risk of a near-singular $\varvec{ \Omega }$ matrix, especially if the moments are strongly mutually (linear) dependent. Calculation of the GMM-estimator with additional external moment functions often includes unknown population moments, like $E({{\textbf {x}}})$ or $\sigma ^2_y$ (see Table 1), which may be replaced by the corresponding sample moments. However, $\varvec{\Omega }_R$ and $\varvec{\Omega }_h$ may in addition be functions of unknown $\sigma ^2$ or $\varvec{\beta }_0$, as can be seen in Table 1. Hence, the externally informed GMM-estimator is calculated iterating over the following steps until convergence: First estimate the model using the ordinary least squares approach without external moments to get ${\hat{\sigma }}^2$ and $\hat{\varvec{\beta }}$ and then estimate $\hat{\varvec{\beta }}_{\textrm{ex}}$ based on the estimates from the former step.

Statistical inference with a GMM-estimator can be based on the Wald test, which simplifies to a t-test if single regression coefficients are tested and its approximative normality can be used to construct confidence intervals (Cameron & Trivedi, 2005). However, in small samples or when dealing with complex models, it is sometimes better to use a bootstrap method (Cameron & Trivedi, 2005; Spiess et al., 2019, p. 177).

As this approach combines data from different sources, one should take into account the issues arising in meta-analyses in general. The *Cochrane Handbook for Systematic Reviews of Interventions* (Higgins et al., 2019) and the PRISMA statement (Page et al., 2021) should be considered to select proper sources of external information, which are as up-to-date and as close as possible to the same population, method and design of the study one wishes to use the externally informed model in. This is important because a core regularity condition of the GMM is that the expected values of the moment functions are zero, which can be violated, if the external moment and the data were taken from different populations. As a possible approach to deal with this compatibility issue, the GMM framework incorporates the Sargan–Hansen test to test if the overidentification due to the additional moment conditions causes a ${\hat{Q}}_n(\hat{\varvec{\theta }}_{\textrm{ex}})$ significantly larger than 0 (Hansen, 1982; Sargan, 1958). Another option to test for incompatibility especially in linear regression models is the Durbin–Wu–Hausman test (Hausman, 1978), as it compares two estimators of the same parameter. We will take a different approach here, as we will instead relax the assumption of correct external point-values to intervals containing the true value.

## Robustness due to Interval Probability

External information is only an estimate itself and thus prone to uncertainty. A classical approach to analyze and prevent the issues of misspecification and thus misleading inferences is to use robust models (Huber, 1981). Hence, it is important to use techniques to robustify the estimation of the externally informed model. In this paper, we will adopt an approach based on the theory of imprecise probabilities due to Weichselberger (2001), that is capable of dealing with probabilistic and non-probabilistic uncertainty, not depending on a fully specified stochastic model. The advantage is that instead of distributional assumptions we only need bounds for the true external values. It would be possible to model the uncertainty in the external information within a probabilistic, e.g., a Bayesian, framework. However, this framework would replace uncertainty in the external information by assuming an additional parametric model of its estimation process in form of precise prior distributions. Moreover, it is not straightforward to represent only certain distributional aspects (moments) within a Baysian approach, e.g., the external information $100=E(y)=E({{\textbf {x}}})^T\varvec{\beta }_0$ presented in Sect. 2.

### Externally Informed Models Based on Interval Information

Assume that $I_{\textrm{ex}}$ is an interval containing the true value of an unknown external moment. Hence every value in the interval could be the true one. To illustrate a possible way to construct an $I_{\textrm{ex}}$, we use our earlier example. In our application example, we have a $95\%$ confidence interval of [0.39, 0.44] for the correlation between fluid intelligence and mathematical skills (Peng et al., 2019). This is, of course, an interval that includes the true value only with a positive probability, but not with certainty. However, combining this confidence interval with the results of other studies on this or a similar correlation, and thus possibly widening the interval, the resulting interval serves as a subjective, rough approximation for $I_{\textrm{ex}}$. We illustrate the use of this technique in Sect. 6.

In this section, we discuss another way of constructing $I_{\textrm{ex}}$. Regarding the estimated depression prevalence of 0.157 in Steffen et al. (2020), we know that $87\%$ of the population has been investigated. Thus, we can construct an interval by the technique proposed, e.g., in Manski (1993, 2003), Manski and Pepper (2013), Cassidy and Manski (2019). The two extreme cases that could occur are that no person of the $13\%$ unobserved individuals has a depression and on the other extreme that all of these individuals have a depression. As $87\%$ of 0.157 is 0.137, we get the interval [0.137, 0.267] for the prevalence. The advantage of such intervals is that they completely compensate for the missing values without any further assumptions. Having available an interval for the external information, one can adopt a technique denoted as cautious data completion proposed by Augustin et al. (2014, p. 182) to determine based on $I_{\textrm{ex}}$ the sets of possible values for the estimator itself and its variance estimator. In our setting, this amounts to evaluating the estimator for the externally informed linear model and its variance estimator from Theorem 1 traversing $I_{\textrm{ex}}$. This leads to a set ${\mathcal {B}}_{\textrm{ex}}$ of possible parameter estimates and a set ${\mathcal {V}}_{\textrm{ex}}$ of possible variance estimates. These sets of estimates are compact and connected in the strict mathematical sense, since both estimators are continuous functions on the external interval.

#### F-Probability

Interval-based inferences can be justified by adopting the concept of F-probabilities (Augustin, 2002; Weichselberger, 2000).

##### Definition 3

(Augustin 2002) Let $\Omega$ be a set and ${\mathcal {A}}$ be a $\sigma$-algebra on $\Omega$. Further, let ${\mathcal {K}}(\Omega ,{\mathcal {A}})$ be the set of all probability measures on $(\Omega ,{\mathcal {A}})$. Then, a set-valued function $F(\cdot )$ on ${\mathcal {A}}$ is called *F-probability* with structure ${\mathcal {M}}$, if

1. there are functions $L(\cdot ), U(\cdot ): {\mathcal {A}} \rightarrow [0,1]$ such that for every event $A \in {\mathcal {A}}$ it holds that $L(A) \le U(A)$ and $F(\cdot )$ has the form
  $$\begin{aligned} F(\cdot ): \quad&{\mathcal {A}} \rightarrow \{[a,b] \, | \, a,b \in [0,1] \text { and } a\le b \} \\&A \mapsto F(A) := [L(A),U(A)] \text { for every event } A \in {\mathcal {A}}, \end{aligned}$$
2. the set ${\mathcal {M}}:= \{P(\cdot ) \in {\mathcal {K}}(\Omega ,{\mathcal {A}}) \, | \, L(A) \le P(A) \le U(A), \text { for all } A \in {\mathcal {A}} \}$ is not empty,
3. for all events $A\in {\mathcal {A}}$ it holds that $\inf _{P(\cdot )\in {\mathcal {M}}} P(A) =L(A)$ and $\sup _{P(\cdot )\in {\mathcal {M}}} P(A) =U(A)$.

For most applications, it is sufficient to restrict to the case $\Omega =\mathbb {R}^d$ and let ${\mathcal {A}}$ be the corresponding Borel $\sigma$-algebra. F-probabilities are best understood as a representation of a “continuous” set of probability measures. For example, consider all normal distributions with a variance of 1 and a mean between $-0.5$ and 0.5. If we consider all these distributions as possible true distributions for a random variable *X* and evaluate an event in terms of its probability, we obtain a set of possible probability values. Consider the event $A=\{X \le 0 \}$, its possible probability ranges from 0.3085 (for mean 0.5) to 0.6915 (for mean $-0.5$) and thus $P(A) \in F(A):= [0.3085,0.6915]$. If this procedure is performed for all $A \in {\mathcal {A}}$, the resulting $F(\cdot )$ is an F-probability. In general, given any nonempty set ${\mathcal {P}}$ of probability measures, one can construct the narrowest F-probability containing ${\mathcal {P}}$ by defining $F(A):=[\inf _{P \in {\mathcal {P}}}P(A),\sup _{P \in {\mathcal {P}}}P(A)]$ for each event $A \in {\mathcal {A}}$, cf. Remark 2.3. in Augustin (2002). If the intervals *F*(*A*) consist of one element for all *A*, the F-probability simply corresponds to a single probability measure. Thus, it is a natural generalization of the conventional notion of probability, using simultaneously a range of probability measures between a lower bound and an upper bound. An important property of F-probabilities for ensuring robustness is that their structure ${\mathcal {M}}$ (all the probability measures covered by $F(\cdot )$, in the sense of condition 2 in Definition 3) is generally larger than the set ${\mathcal {P}}$ (called pre-structure) of probability measures used to construct them, since the structure is closed under convex combinations (Augustin, 2002). For two probability measures *P* and *Q*, this follows by the basic inequality that for all $0\le \epsilon \le 1$ and $A \in {\mathcal {A}}$ it holds that

$$\begin{aligned} \min (P(A),Q(A) ) \le \epsilon P(A) + (1-\epsilon ) Q(A)\le \max (P(A),Q(A)). \end{aligned}$$

For example, convex combinations of normal distributions are not themselves normally distributed and include skewed and bimodal distributions. This illustrates that robustness with respect to distributional assumptions increases compared to using normal distributions alone. Unlike other concepts that reflect uncertainty about probability measures, such as triangular numbers (fuzzy numbers), there is no preference for one distribution over another caused by weighting functions or possibility distributions. This agnosticism regarding the true distribution also covers deterministic ambiguity to some extent. For instance, in our example, a deterministic alteration of $\mu$ over time, where $\mu (t)\in [-0.5,0.5]$ for all *t*, like $\mu (t)=0.5\sin (t)$, would still be covered by the F-probability at any time *t* because the F-probability covers the range of $\mu (t)$. In applied research, the exact form of deterministic variation of $\mu$ is typically unknown, but if its bounds are known to lie within an interval, the F-probability based on this interval would account for it. Of course, these advantages come at the cost of greater conservatism than using a single probability distribution.

In our framework, the assumption of knowing the true moment value can be relaxed to assuming that an interval is known containing the unknown true moment value. As the GMM-estimator is asymptotically normally distributed for the true value of the external moment, we asymptotically get a pre-structure consisting of all normal distributions for estimator $\hat{\varvec{\beta }}_{\textrm{ex}}$ with expected value inside ${\mathcal {B}}_{\textrm{ex}}$ and with variance inside ${\mathcal {V}}_{\textrm{ex}}$. This pre-structure is guaranteed to contain the normal distribution based on GMM asymptotics, since the true external moment value is assumed to be in $I_{\textrm{ex}}$. Therefore, for each event, the probability assigned to an event by this true normal distribution will lie between the lower and upper bounds assigned to that event by $F(\cdot )$, possibly leading to more conservative but valid statistical inference. Based on this pre-structure, we get an F-probability. Statistical inference based on F-probabilities is done by treating the probability intervals as a whole, e.g., by interval arithmetic. We demonstrate this principle by constructing an equivalent to confidence intervals in the context of F-probabilities in the next section.

#### Confidence Intervals for the Externally Informed Model Under F-Probabilities

The construction of confidence intervals (point-CIs) is in general not possible in the framework of F-probabilities, because instead of a single probability value lower and upper bounds are assigned to an event. One possibility, however, is to use the union of all possible point-CIs traversing $I_{\textrm{ex}}$. The idea to calculate unions of intervals already has been investigated for Bayesian highest density intervals in an imprecise probability setting by Walter and Augustin (2009). Let $\hat{\varvec{\theta }}_{e,j}$ be the *j*-th entry of the externally informed GMM-estimator $\hat{\varvec{\theta }}_{\textrm{ex}}$ using external value *e*, we define the $(1-\alpha ) \cdot 100\%$ confidence union for $\varvec{\theta }_{j}$ to be

$$\begin{aligned} \bigcup {\text {CI}}_{1-\alpha } := \left[ \inf _{e \in I_{\textrm{ex}}} [ \hat{\varvec{\theta }}_{e,j} - t_{1-\frac{\alpha }{2},n-p} \sqrt{\widehat{\text {Var}}(\hat{\varvec{\theta }}_{e,j})}] , \sup _{e \in I_{\textrm{ex}}} [\hat{\varvec{\theta }}_{e,j} + t_{1-\frac{\alpha }{2},n-p} \sqrt{\widehat{\text {Var}}(\hat{\varvec{\theta }}_{e,j})}] \right] \end{aligned}$$

Because the true external moment value is in $I_{\textrm{ex}}$, the borders of the point-CI constructed via the true moment value lie between the infimum and the supremum of the lower and upper borders, respectively, of all point-CIs on $I_{\textrm{ex}}$. Therefore, $\bigcup {\text {CI}}_{1-\alpha }$ covers the point-CI constructed via the true moment value. The asymptotic normal distribution of $\hat{\varvec{\beta }}_{\textrm{ex}}$ at the true value of the external moment implied by the asymptotic properties of GMM-estimators described in Sect. 2 ensures that the confidence union covers the true parameter asymptotically with probability at least $1-\alpha$.

An approximation of the confidence union can be calculated using grid search traversing $I_{\textrm{ex}}$. If the point-CIs used to construct $\bigcup {\text {CI}}_{1-\alpha }$ differ, then the resulting interval is wider than every of these point-CIs. This demonstrates that the positive effect of the variance reduction (a shorter CI) can be reversed by the length of $I_{\textrm{ex}}$. The reason is that a broader $I_{\textrm{ex}}$ increases the set over which infimum and supremum are taken, possibly expanding $\bigcup {\text {CI}}_{1-\alpha }$. However, we will show in a simulation study in Sect. 5 that in some cases it is possible to get a $\bigcup {\text {CI}}_{1-\alpha }$ shorter than the $(1-\alpha )$ confidence interval based on the OLS multiple linear regression. Hence, the variance reduction can compensate the broadening of $\bigcup {\text {CI}}_{1-\alpha }$ introduced by $I_{\textrm{ex}}$. Finally, using $\bigcup {\text {CI}}_{1-\alpha }$ strengthens the robustness through the F-probability, on which $\bigcup {\text {CI}}_{1-\alpha }$ is based, as it also includes, e.g., bimodal and skewed distributions.

## A Simulation Study

### Settings

To test the externally informed GMM approach for multiple linear models in small samples, we conducted two simulation studies. The first setting illustrates possible variance reduction if correctly specified external moments are used and shows that the usage of small external moment intervals can lead to confidence unions that may even be shorter than the OLS confidence interval. In the second setting, we focus on misspecified external information and non-normal errors. In this case, it is interesting to see, if inferences are still valid and whether the effects of the variance reduction illustrated in the first setting still occur. The simulation script was written and executed in R version 4.2.1 (R Core Team, 2022), the script can be found in the supplementary materials online. The function interval_gmm() implements the calculation of intervals of estimators and of their standard deviation, as well as confidence interval unions. In both settings, we used an intercept ($x_1=1$), a normally distributed variable $x_2 \sim N(2,4)$ and a binary variable distributed according to Bernoulli distribution $x_3 \sim {\text {Bernoulli}}(0.4)$ as explanatory variables. The response variable was generated according to $y=x_1+0.5x_2+2x_3+\epsilon$, where $\epsilon \sim N(0,9)$ in the first setting. In the second setting, the errors were generated by affine transformation of a $\chi _1^2-$distributed random sample, so that its mean is 0 and its variance is 9. The settings were selected, so that all required moments can easily be calculated, which is done before the simulations. The ratio of explained variance to total variance was $1-9/\text {Var}(y)=1-9/10.96=0.178$ a value which is similar to often reported values in psychological research. This amounts to a relatively high error variance, a factor for possibly large variance reduction for some external moments (see Sect. 3).

Different moments have different scales, so a similar interval width of $I_{\textrm{ex}}$ does not imply similar “sharpness” of the external information across scales. To create intervals for the external information, that are comparable across the different scales of the external moments, we have used external intervals where the ratio of half their width to their center is the same for all external moments in each setting. It should be noted that this technique is different from the design techniques discussed in Sect. 4.1. The reason for this difference is that the simulation study aims to compare the different moments in terms of their effectiveness and statistical validity in a context where the $I_{\textrm{ex}}$ are comparable in magnitude and contain the true value. To motivate this, one could compare the given ratio to the coefficient of variation. For the standard IQ-scale, the coefficient of variation is $15/100=0.15$. For the first setting, we arbitrarily chose a ratio of 0.1 to represent somewhat more precise external information than one standard deviation in the IQ-scale around the center. For the second setting, we have chosen a ratio of 0.3 to represent a radius of two standard deviations in the IQ-scale and thus an approximate confidence interval width that takes the IQ-scale as a basis. In the first setting, we created intervals that were symmetrical around the true external value. Hence, if the true external value was *e*, then the interval was $I_{\textrm{ex}}=[0.9e, 1.1 e]$. In the second setting, we first multiplied all true external moment values by 1.3. Since none of these true external values were equal to zero, this resulted in misspecified point values. These misspecified values were used as external point values during the simulation to test the sensitivity of the externally informed model based on point information. The constant 1.3 was again chosen arbitrarily and leads to a relative bias of $30\%$. Then, as in the first setting we generated a symmetric interval around the misspecified value. If *e* again denotes the true external value, $0.7\cdot 1.3e=0.91e$ was the lower limit and $1.3\cdot 1.3e=1.69e$ the upper limit of $I_{\textrm{ex}}$, i.e. $I_{\textrm{ex}}=[0.91e,1.69e]$, which contains the true value *e*. As for sensitivity, tests with center width ratio and misspecification values similar to 0.1, 0.3 and 1.3 gave similar results.

Sample sizes *n* chosen are 15, 30, 50, 100. The moments used are those listed in Table 2 for both, $x_2$ and $x_3$. Given the results in Sect. 3, the expected relative variance reductions were calculated to check if these settings are capable of providing enough variance reduction. For every moment condition in each setting we run 500 simulations. Only single moment conditions were used.

In a first step, all explanatory variables were generated and *y* was calculated as described above. In the second step, $\hat{\varvec{\beta }}_{\textrm{ex}}$ and $\widehat{\text {Var}}(\hat{\varvec{\beta }}_{\textrm{ex}})$ were calculated according to the following two-step GMM algorithm:

1. Calculate $\hat{\varvec{\beta }}$ and ${\hat{\sigma }}^2$ via the classical OLS method
2. Determine $\hat{\varvec{ \Omega }}_R$, ${\hat{\omega }}_h$ and $\hat{\varvec{\beta }}_{\textrm{ex}}$ based on $\hat{\varvec{\beta }}$ and ${\hat{\sigma }}^2$
3. Recalculate ${\hat{\sigma }}^2,\hat{\varvec{ \Omega }}_R$ and ${\hat{\omega }}_h$ based on $\hat{\varvec{\beta }}_{\textrm{ex}}$
4. Update $\hat{\varvec{\beta }}_{\textrm{ex}}$ and calculate $\widehat{\text {Var}}(\hat{\varvec{\beta }}_{\textrm{ex}})$

Then, $95\%$ confidence intervals were calculated based on $\hat{\varvec{\beta }}_{\textrm{ex}}$ and its estimated variance, using a t-distribution with $n-3$ degrees of freedom. Let ${\hat{\beta }}_{\textrm{ex}}$ be one element of $\hat{\varvec{\beta }}_{\textrm{ex}}$, then it’s $95\%$ confidence interval is

$$\begin{aligned} {\text {CI}}_{0.95}=\left[ {\hat{\beta }}_{\textrm{ex}}-t_{n-3,0.975}\sqrt{\widehat{\text {Var}}({\hat{\beta }}_{\textrm{ex}})},{\hat{\beta }}_{\textrm{ex}}+t_{n-3,0.975}\sqrt{\widehat{\text {Var}}({\hat{\beta }}_{\textrm{ex}})}\right] . \end{aligned}$$

To calculate $\bigcup {\text {CI}}_{0.95}$ a grid search algorithm was adopted. First we determined 101 equidistant points in the given $I_{\textrm{ex}}$ (including the bounds of the interval). The number 101 was chosen after some preliminary tests of the algorithm as a compromise between precision and computing time. Then we traversed these grid points calculating ${\hat{\beta }}_{\textrm{ex}}$ and $\widehat{\text {Var}}({\hat{\beta }}_{\textrm{ex}})$ using the two step procedure from above at each point. Comparing the bounds of the CIs sequentially, the minimal lower and maximal upper CI bounds on the grid points were determined and served as approximation for the bounds of $\bigcup {\text {CI}}_{0.95}$.

### Results

As criteria to evaluate the statistical inferences, we calculated the mean $\bar{\hat{\varvec{\beta }}}_{\textrm{ex}}$ of the estimates $\hat{\varvec{\beta }}_{\textrm{ex}}$ and their variances $\text {Var}(\hat{\varvec{\beta }}_{\textrm{ex}})$ over 500 simulations. The latter will be compared to the corresponding means of estimated variances, $\overline{ \widehat{\text {Var}}(\hat{\varvec{\beta }}_{\textrm{ex}})}$. To evaluate possible variance reduction for $\beta _j$, the mean ratio of variance reduction to OLS-variance, ${\hat{\Delta }}_j:=\overline{[\widehat{\text {Var}}(\hat{\varvec{\beta }}_{OLS})-\widehat{\text {Var}}(\hat{\varvec{\beta }}_{\textrm{ex}})]_{(j,j)}/[ \widehat{\text {Var}}(\hat{\varvec{\beta }}_{OLS})]_{(j,j)}}$, will be considered. In addition, the actual coverage is calculated over simulations. For given $\alpha =0.05$ and 500 simulations, the actual coverage should be between 0.93 and 0.97 for the point-valued moments (Spiess, 1998) and equal to or greater than 0.93 for the external moment intervals, as the confidence union is used to calculate the coverage in this case. Finally, $|{\text {CI}}|:= \overline{{\overline{{\text {CI}}}}_{0.95}-{\underline{{\text {CI}}}}_{0.95}}$ and $|\bigcup {\text {CI}}|:=\overline{\overline{\bigcup {\text {CI}}}_{0.95}-\underline{ \bigcup {\text {CI}}}_{0.95}}$ were computed. They can be compared to the OLS-CI-length to evaluate the possible precision gains or losses.

#### Results for the Correctly Specified Setting

The detailed results for sample size $n=15$ are presented in Table 3, while the results for the other sample sizes are given in Tables 7 to 9 in the supplementary materials. Consistent with the theory in Sect. 3, the use of the moment $E(x_2)$ had no effect on the variances, neither for the correctly specified nor for the misspecified setting, and estimation results were equal to OLS estimation results. The corresponding results are presented for comparison. For all moments except $E(y^2)$ and $\sigma ^2_y$ both the coverages for the point valued moments as well as the coverages for the external intervals exceeded 0.93. The coverages for $\sigma ^2_y$ were in the valid range only for $n=100$, while for $E(y^2)$ they were in the valid range already for $n=50$. The undercoverage for sample sizes below $n=100$ can be explained by the skewness of the distributions of their sample moment functions in small samples caused by the quadratic terms $y^2$, leading to higher sample size required for the asymptotic results to be applicable. Using confidence unions only reduced these required sample sizes to $n=50$ and $n=30$, respectively, showing that high skewness is also problematic for $\bigcup {\text {CI}}$-based coverage in small samples.

**Table 3:** Results of the simulations with correctly specified external moments for sample size $n=15$.

Moments	$\beta _j$	$\bar{{\hat{\beta }}}_{\textrm{ex}}$	Var(${\hat{\beta }}_{\textrm{ex}}$)	$\overline{ \widehat{\text {Var}}({\hat{\beta }}_{\textrm{ex}})}$	${\hat{\Delta }}_j$	Cov	Cov_I_	|${\text {CI}}$|	|$\bigcup {\text {CI}}$|
$E(x_2)$	$\beta _1$	1.045	1.929	1.968	0	0.944	0.944	5.866	5.866
= OLS	$\beta _2$	0.499	0.205	0.211	0	0.950	0.950	1.909	1.909
	$\beta _3$	1.955	3.422	2.976	0	0.940	0.940	7.292	7.292
*E*(*y*)	$\beta _1$	1.048	1.453	1.593	0.217	0.942	0.964	5.215	5.639
$E(x_2y)$	$\beta _2$	0.560	0.174	0.177	0.169	0.950	0.956	1.740	1.832
$\sigma _{x_2,y}$	$\beta _1$	0.802	1.478	1.456	0.239	0.954	0.954	5.054	5.222
	$\beta _2$	0.625	0.096	0.080	0.616	0.960	0.978	1.153	1.237
$\rho _{x_2,y}$	$\beta _1$	0.928	1.405	1.418	0.258	0.944	0.948	4.984	5.141
	$\beta _2$	0.562	0.069	0.074	0.639	0.976	0.984	1.110	1.188
$\beta _{x_2,y}$	$\beta _1$	0.967	1.263	1.414	0.255	0.960	0.964	4.982	5.131
	$\beta _2$	0.543	0.054	0.072	0.633	0.986	0.986	1.107	1.180
$E(x_3y)$	$\beta _3$	2.150	2.745	2.380	0.192	0.936	0.946	6.525	6.837
$\sigma _{x_3,y}$	$\beta _1$	0.959	1.552	1.704	0.141	0.964	0.966	5.433	5.561
	$\beta _3$	2.202	0.783	0.949	0.689	0.976	0.976	3.952	4.273
$\rho _{x_3,y}$	$\beta _1$	0.977	1.659	1.694	0.148	0.960	0.960	5.415	5.541
	$\beta _3$	2.136	0.888	0.919	0.699	0.964	0.970	3.891	4.208
$\beta _{x_3,y}$	$\beta _1$	0.942	1.545	1.700	0.143	0.964	0.970	5.426	5.559
	$\beta _3$	2.182	0.692	0.908	0.698	0.976	0.980	3.888	4.213
$E(y^2)$	$\beta _1$	1.072	1.965	1.935	0.018	0.914	0.936	5.807	5.950
	$\beta _2$	0.507	0.207	0.204	0.031	0.942	0.946	1.875	1.929
	$\beta _3$	1.995	3.414	2.871	0.030	0.916	0.930	7.164	7.367
$\sigma ^2_y$	$\beta _1$	0.905	1.951	1.708	0.142	0.920	0.928	5.434	5.667
	$\beta _2$	0.540	0.228	0.184	0.148	0.896	0.910	1.766	1.839
	$\beta _3$	2.092	3.646	2.584	0.151	0.858	0.878	6.725	7.005

The expressions $\bar{{\hat{\beta }}}_{\textrm{ex}}$, Var(${\hat{\beta }}_{\textrm{ex}}$), $\overline{ \widehat{\text {Var}}({\hat{\beta }}_{\textrm{ex}})}$, ${\hat{\Delta }}_j$,|${\text {CI}}$| and |$\bigcup {\text {CI}}$| are defined in the beginning of Sect. 5.2. The results for the moment $E(x_2)$ are equivalent to the OLS results. Cov is the coverage for the external point value and $\text {Cov}_I$ symbolizes the coverage for the confidence interval union based on the external interval. Only the affected coefficients are reported per moment. The true values are $\beta _1=1$, $\beta _2=0.5$ and $\beta _3=2$.

For $\beta _{x_j,y}$ with $j=2,3$, the coverage for $\beta _{j}$ was in many cases above 0.97 (up to 0.994) for all *n*. This was also the case, though not as pronounced, when the external information about the covariance between $x_j$ and *y* was used. The reason for this is that the variances were mostly overestimated in these cases, as can be seen in Tables 3 and 4 as well as in Tables 7 to 12 in the supplementary materials by the fact that $\overline{ \widehat{\text {Var}}({\hat{\beta }}_{\textrm{ex}})}$ was larger than Var(${\hat{\beta }}_{\textrm{ex}}$) for the respective $\beta _j$. Although variances are overestimated, the true and estimated variances nevertheless tend to be smaller than the variance of the OLS-estimators. Thus, inferences still tend to be more precise, suggesting a possible relationship with superefficiency (Bahadur, 1964).

As shown in Sect. 3, the relative variance reduction for each estimator of $\beta _j$, reported in column ${\hat{\Delta }}_j$ of Table 3 as well as Tables 7 to 9 in the supplementary materials, did not change significantly under the various conditions over the different sample sizes realized. The smallest relative variance reduction per $\beta _j$ was attained by using the external information $E(y^2)$, ranging from 0.018 to 0.059, followed by $\sigma ^2_y$ with a maximal relative variance reduction of 0.180. The largest relative variance reduction was attained by using the covariance, the correlation and $\beta _{x_j,y}$ regarding $\beta _{j}$, ranging from 0.633 to 0.734 for $j=2$ as well as from 0.698 to 0.857 for $j=3$. For all other moments the values varied between 0.169 and 0.294, see Table 3 and Tables 7 to 9 in the supplementary materials.

These variance reductions translated for all moments directly into a reduction of the length of the confidence interval for the external point value. For the external interval, the length of the union of the confidence intervals is always greater than the one derived from a single external point. These differences increase with larger samples, as the variance estimator decreases with increasing sample sizes, while it can be seen from the formulas in Theorem 1 that the interval for ${\hat{\beta }}_{\textrm{ex}}$ is only affected by the difference between the estimators and the true values of $\varvec{ \Omega }_{R}$ and $\varvec{ \Omega }_{h}$, not directly by *n*. Finally, with regard to $|\bigcup {\text {CI}}|$ compared to $|{\text {CI}}|$ the results imply that at the sample sizes 15 and 30, using any moment except $E(y^2)$ resulted in a shorter confidence interval union than the OLS confidence interval. For $n=50$, this was the case for all moments except $E(y^2)$, *E*(*y*) and $\sigma ^2_y$. Finally, for $n=100$, only the moments $\sigma _{x_2,y}$, $\rho _{x_2,y}$, $\beta _{x_2,y}$, $\sigma _{x_3,y}$, $\rho _{x_3,y}$ and $\beta _{x_3,y}$ resulted in shorter confidence unions than point-CIs. This can be explained by the constancy of $I_{\textrm{ex}}$ while *n* increases. There is always an interval inside $\bigcup {\text {CI}}$ which does not vanish for large *n*, while $|{\text {CI}}|$ converges to 0.

#### Results for the Misspecified Setting

The detailed results for sample size $n=50$ are presented in Table 4, while the results for the other sample sizes are given in Tables 10 to 12 in the supplementary materials. The coverage rates using the point-valued moments illustrate the expected sensitivity of the models due to misspecification. Even at $n=15$ more than half of the coverage rates are below 0.93, although in most cases they are still above 0.9. The severeness increases with increasing *n*: For $n=30$ only five coverage rates are in the acceptable range of at least 0.93. As seen in Table 4 for $n=50$ the coverage is as low as 0.586 in the worst case for $\beta _3$ if $\sigma _{x_3,y}$ is used. Finally, for $n=100$ all coverage rates are invalid, see Table 12 in the supplementary materials. Except for the moments $E(y^2)$ and $\sigma ^2_y$, this is corrected by the union of confidence intervals based on the external interval, since all coverage rates in these cases are above 0.93, except the one for $\beta _1$ using $\sigma _{x_2,y}$ while $n=15$. Like in the correctly specified setting, there are considerably larger coverage rates for the moments $\beta _{x_j,y}$ and lower coverage rates for $\sigma ^2_y$ or $E(y^2)$ even in the cases $n=30$ and $n=15$. The explanations for these over- and undercoverages are the same as for the correctly specified case in Sect. 5.2.1. However, only the use of covariance, correlation or $\beta$ for $x_j$ and *y* for $j=2,3$ resulted in narrower confidence unions as compared to OLS confidence intervals, not the use of other moments. Regarding $\beta _{j}$ for $j=2,3$ this is the case for every *n*, regarding $\beta _1$ this is only the case for $n=15$. We conclude that the use of external intervals for covariances, correlations or $\beta$ not only corrects low coverage rates due to misspecified point values for external moments, but can also lead to narrower (unions of) confidence intervals.

**Table 4:** Results of the simulations with misspecified external moments for sample size $n=50$.

Moments	$\beta _j$	$\bar{{\hat{\beta }}}_{\textrm{ex}}$	Var(${\hat{\beta }}_{\textrm{ex}}$)	$\overline{ \widehat{\text {Var}}({\hat{\beta }}_{\textrm{ex}})}$	Cov	Cov_I_	|CI|	|$\bigcup {\text {CI}}$|
$E(x_2)$	$\beta _1$	1.012	0.440	0.509	0.936	0.936	2.778	2.778
= OLS	$\beta _2$	0.488	0.041	0.049	0.960	0.960	0.865	0.865
	$\beta _3$	2.042	0.751	0.803	0.954	0.954	3.494	3.494
*E*(*y*)	$\beta _1$	1.636	0.412	0.376	0.782	0.992	2.397	4.032
$E(x_2y)$	$\beta _2$	0.615	0.031	0.040	0.924	0.984	0.779	1.062
$\sigma _{x_2,y}$	$\beta _1$	0.733	0.307	0.380	0.912	0.954	2.409	2.922
	$\beta _2$	0.627	0.016	0.016	0.864	0.984	0.496	0.747
$\rho _{x_2,y}$	$\beta _1$	0.756	0.243	0.380	0.952	0.968	2.401	2.914
	$\beta _2$	0.617	0.019	0.016	0.880	0.996	0.494	0.744
$\beta _{x_2,y}$	$\beta _1$	0.768	0.271	0.376	0.926	0.960	2.397	2.901
	$\beta _2$	0.610	0.008	0.015	0.948	0.998	0.488	0.735
$E(x_3y)$	$\beta _3$	2.505	0.486	0.622	0.934	0.982	3.095	4.165
$\sigma _{x_3,y}$	$\beta _1$	0.827	0.341	0.413	0.930	0.966	2.502	3.026
	$\beta _3$	2.549	0.116	0.139	0.586	1.000	1.445	2.751
$\rho _{x_3,y}$	$\beta _1$	0.830	0.291	0.413	0.956	0.978	2.498	3.025
	$\beta _3$	2.525	0.341	0.138	0.642	0.978	1.434	2.739
$\beta _{x_3,y}$	$\beta _1$	0.822	0.346	0.412	0.922	0.966	2.499	3.030
	$\beta _3$	2.537	0.092	0.135	0.618	1.000	1.428	2.740
$E(y^2)$	$\beta _1$	1.136	0.512	0.513	0.914	0.944	2.802	3.151
	$\beta _2$	0.558	0.053	0.049	0.894	0.948	0.863	1.011
	$\beta _3$	2.318	0.846	0.790	0.894	0.958	3.477	4.093
$\sigma ^2_y$	$\beta _1$	0.512	0.894	0.433	0.698	0.828	2.543	3.361
	$\beta _2$	0.626	0.067	0.044	0.754	0.896	0.806	1.029
	$\beta _3$	2.597	1.054	0.697	0.750	0.896	3.234	4.167

The expressions $\bar{{\hat{\beta }}}_{\textrm{ex}}$, Var(${\hat{\beta }}_{\textrm{ex}}$), $\overline{ \widehat{\text {Var}}({\hat{\beta }}_{\textrm{ex}})}$,|${\text {CI}}$| and |$\bigcup {\text {CI}}$| are defined in the beginning of Sect. 5.2. The results for the moment $E(x_2)$ are equivalent to the OLS results. Cov is the coverage for the external point value and $\text {Cov}_I$ symbolizes the coverage for the confidence interval union based on the external interval. Only the affected coefficients are reported per moment. The true values are $\beta _1=1$, $\beta _2=0.5$ and $\beta _3=2$.

## Application

To illustrate the possible benefits of using external information in a linear model, we reanalyze a dataset of Pluck and Ruales-Chieruzzi (2021), who investigated the estimation of premorbid intelligence based on lexical reading tasks in Ecuador. We will focus on their Study 2. Since the purpose of this analysis is to illustrate the proposed use of external information, we will only shortly sketch the theoretical background of the study. For a more detailed description, see Pluck and Ruales-Chieruzzi (2021). The dataset was downloaded from PsychArchives (Pluck, 2020a).

To quantify the cognitive impairment of patients, it is necessary to have an accurate baseline estimate observed in the premorbid state (Pluck & Ruales-Chieruzzi, 2021). As psychometric intelligence tests can be too long or cumbersome for elderly people with emerging cognitive impairments, it is important to have short, yet reliable tests for general intelligence. It is argued in Pluck and Ruales-Chieruzzi (2021) that vocabulary has a high positive correlation with general intelligence, hence using short lexical tests could be helpful to estimate general intelligence. Following Cattell’s classical theory, general intelligence can be divided into fluid and crystallized intelligence (Cattell, 1963). In this context, the variance reduction property of the externally informed linear model could provide an asymptotically unbiased estimate with higher precision than the estimates in Pluck and Ruales-Chieruzzi (2021), because external information about the correlation of general, fluid or crystallized intelligence and lexical tests is available. Although the different factors of intelligence are not identical, combining external information about them leads to a broader and thus more reliable external interval than using information about general intelligence alone, as the correlation between lexical tasks and fluid or crystallized intelligence may be lower or higher than for general intelligence.

In their Study 2 Pluck and Ruales-Chieruzzi (2021) used a Spanish, validated seven-subtest version of the Wechsler Adult Intelligence Scale in the 4th edition (WAIS-IV) (Meyers et al., 2013) to measure general intelligence, as well as three lexical tests, the Word Accentuation Test (WAT) in Spanish (Del Ser et al., 1997), the Stem Completion Implicit Reading Test (SCIRT) (Pluck, 2018) and the Spanish Lexical Decision Task (SpanLex) (Pluck, 2020b). The sample consists of 106 premorbid participants without neurological illness. As one participant has not completed the WAT, this person was excluded from the analysis regarding the WAT score. Simple linear regression models with the WAIS-IV as dependent and the lexical tests as independent variable, respectively, were conducted to determine the percentage of explained variance and to test the predictability of general intelligence through every single test. Therefore, the sample was randomly divided into two halves; hence, the net sample size for the linear regression models was 53 as the other half was used to test the prediction based on the regression models. We compared the widths of the $95\%$ confidence intervals for the parameters of these regression models to the widths of the $95\%$ confidence unions resulting from externally informed versions of the linear models. Because the OLS estimation does not account for heteroscedastic errors, which are common in practice, the standard errors are often too small (White, 1980). To correct for heteroscedasticity, we have computed robust standard errors of type HC3 using the package *sandwich* (Zeileis, 2004; Zeileis et al., 2020). Since the dependent variable is the WAIS-IV, an intelligence test with calibration sample, we calculated $E(y)=100$. In the simulation study using the external information about $\rho$ was found to lead to high variance reduction. Hence, by reviewing the literature, we identified the upper bound for the correlation between general intelligence and lexical tasks to be .85. This value was reported as correlation between WAT and the vocabulary scale of the Wechsler Adult Intelligence scale in Burin et al. (2000). A lower bound for the correlation between general intelligence and lexical tasks was found using the meta-analysis of Peng et al. (2019) or the study of Pluck (2018). Pluck (2018) argued based on a couple of studies that the correlation of general intelligence and lexical skills is typically higher than .70. In the meta-analysis of Peng et al. (2019), the reported $95\%$ confidence interval for the correlation of fluid intelligence and reading is [0.36, 0.39]. To compare the results, both sources were used separately, leading to the lower bounds 0.4 and 0.7, where 0.4 is very conservative as it is derived from a correlation including a different variable (fluid intelligence). Together this amounts to the intervals [0.4, 0.85] and [0.7, 0.85], which are adopted for each of the three lexical tests. The confidence unions were calculated in the same way as in the simulations using grid search, but with 10001 grip points instead of 101 and $\hat{\varvec{ \Omega }}_h=\frac{1}{n}\sum _{i=1}^n {{\textbf {h}}}({{\textbf {z}}}){{\textbf {h}}}({{\textbf {z}}})^T$. The details of the analysis can be found in the R script in the online supplements to this article. The results for the interval [0.7, 0.85] are shown in Table 5 and the results for the interval [0.4, 0.85] are in Table 13 in the supplementary materials. First, the results of Pluck and Ruales-Chieruzzi (2021) were recalculated, showing no differences from the results reported in their Study 2. In addition, the corresponding OLS confidence intervals for the parameters were calculated based on the HC3 estimator (see column five of Table 5). Then, estimator and standard error intervals, as well as the unions of confidence intervals, were calculated for the externally informed model. For both [0.4, 0.85] and [0.7, 0.85], the maxima of all standard error intervals were below the respective standard errors calculated for the OLS models of Pluck and Ruales-Chieruzzi (2021). This clearly shows the variance reduction property of the externally informed model and was most pronounced for the SpanLex. For [0.4, 0.85] all estimation intervals included the OLS estimates and all confidence unions were larger than the corresponding OLS confidence intervals, indicating that [0.4, 0.85] is very conservative. For [0.7, 0.85], the estimation interval $[\underline{{\hat{\beta }}_j}, \overline{{\hat{\beta }}_{j}}]$ included the OLS estimator only for the slope and intercept of the regression on SCIRT and the one based on the WAT. In this case, however, all confidence unions overlapped with the OLS-based confidence intervals. Using [0.7, 0.85], for every lexical test, the widths of the confidence unions from the externally informed model were smaller than the confidence intervals from the simple linear regression models, for both slopes and intercepts, except for the intercept of WAT. Since the prediction interval is calculated based on the distribution of parameter estimators, this would lead to shorter prediction intervals for a participant’s general intelligence based on the externally informed model. In addition, the confidence union approach is more robust than OLS confidence intervals with respect to deviations from the assumed normal distribution. Taken together, this amounts to possibly more precise yet robust parameter estimation and prediction, if the external information is correct.

**Table 5:** Results using $\rho _{x,y} \in [0.7,0.85]$ and $E(y)=100$.

*j*	Test	Pluck and Ruales-Chieruzzi			Externally informed estimates		
		${\hat{\beta }}_{j}$	$s({\hat{\beta }}_{j})$	$CI_{0.95}$	$[\underline{{\hat{\beta }}_j}, \overline{{\hat{\beta }}_{j}}]$	$[\underline{s({\hat{\beta }}_j)}, \overline{s({\hat{\beta }}_{j})}]$	$\bigcup CI_{0.95}$
1	SpanLex	54.61	8.864	[37.06, 72.15]	[37.41, 47.15]	[2.373, 2.663]	[32.06, 51.91]
	WAT	62.81	4.701	[53.51, 72.12]	[60.02, 62.89]	[3.587, 3.612]	[52.77, 70.10]
	SCIRT	60.81	4.395	[52.11, 69.51]	[59.01, 61.28]	[3.910, 3.920]	[51.14, 69.13]
2	SpanLex	1.821	0.332	[1.163, 2.480]	[2.068, 2.430]	[0.124, 0.132]	[1.818, 2.696]
	WAT	2.083	0.240	[1.607, 2.559]	[2.041, 2.186]	[0.190, 0.191]	[1.659, 2.568]
	SCIRT	3.292	0.358	[2.583, 4.001]	[3.213, 3.393]	[0.309, 0.310]	[2.592, 4.015]

The third and fourth columns contain the recomputed results of Pluck and Ruales-Chieruzzi (2021) in terms of the OLS regression coefficients ${\hat{\beta }}_j$, where ${\hat{\beta }}_1$ is the intercept and ${\hat{\beta }}_2$ is the slope and the robust standard errors $s({\hat{\beta }}_{j})$ of the coefficients. The (robust) $95\%$ confidence intervals $CI_{0.95}$ for the parameters were computed in addition. The estimator interval $[\underline{{\hat{\beta }}_j}, \overline{{\hat{\beta }}_{j}}]$, the standard error interval $[\underline{s({\hat{\beta }}_j)}, \overline{s({\hat{\beta }}_{j})}]$ and the $95\%$ confidence interval union $\bigcup CI_{0.95}$ are shown as results of the estimation of the externally informed model.

## Discussion

In this paper, we show that incorporating external moments into the GMM framework by using intervals instead of point values can lead to more robust analyses, while a possible variance reduction can prevent the confidence unions from being too wide.

The results of the simulation study for point values show that the variance reduction can be considerable, over $70\%$ using external information about covariances, correlations or $\beta _{x_j,y}$. However if the external moments deviate from the true values, the inferences will be biased, getting worse with increasing sample size. Instead, the use of external intervals often leads to correct inferences. However, the F-probability couldn’t completely correct the undercoverages caused by using the moments $\sigma ^2_y$ as well as $E(y^2)$, though it slightly improved them. The reason for these undercoverages is the skewed distribution induced by $y^2$, indicating a limitation of the distributional robustness in the presence of large deviations from the normal distribution. As these two moments also showed low variance reduction when used, one should thoughtfully decide on basis of their relative variance reduction if one wants to use them in small samples. However, bootstrap methods, like the bias-corrected accelerated bootstrap (Efron & Tibshirani, 1993), could be used instead to try to correct the undercoverage.

For small sample sizes, the use of covariances, correlations, and $\beta _{x_j,y}$, $j=2,3$, leads to variance reduction despite the use of external intervals. However, this was mostly the case for certain entries $\beta _j$ of $\varvec{\beta }$ in this setting, not for all elements in $\varvec{\beta }$. Interestingly, the use of covariances and $\beta _{x_j,y}$, $j=2,3$, still resulted in overcoverage caused by overestimation of the variance. This means that inferences based on these moments would be more conservative than necessary, yet they had the highest variance reduction of all the moments tested, providing an interesting link to the concept of superefficiency (Bahadur, 1964). Further research on the variance estimator is needed to potentially correct for its overestimation.

Taken together, the simulation study showed promising results regarding very small sample sizes as $n=15$, and however, one should still be cautious as the estimators are only proved to be consistent, not unbiased. To be sure that the inference will be valid in the sample at hand, a simulation to test the adopted scenario, i.e., model to be estimated and data set, is advised. In Sect. 6, we showed the applicability of the theoretical results to real data, where for the variable SpanLex the width of the confidence unions was significantly smaller than the width of the corresponding point-CI, if an appropriately small external interval is used. This shows the usefulness of adopting an externally informed model for applied problems.

A possible limitation of GMM is the assumption of the covariance matrix of the external moments being positive definite, which excludes distributions for which the required covariance matrix does not exist, e.g., the Cauchy distribution. Nevertheless, in many psychological applications the variables have a constrained range of values, so that at least the existence of the covariance matrix can be assumed. In general, the applicability of the method is not overtly limited by its assumptions. Another limitation is that the true value of the external moment must be within the external interval. However, this identifiability assumption, or an analogous assumption, exists in other approaches, and it is much weaker than point identifiability. Thus, a more robust use of external information is possible, up to using the full range of possible values, which would definitely lead to a valid, more robust, but also very conservative inference. The construction of the external moment interval in Sect. 6 was based on a rough, subjective approximation. The question of how to construct the external intervals requires further research. In particular, further links to existing techniques for eliciting intervals and preventing overconfidence bias would be important.

An application of the theory to generalized linear models or multi-level models is of inherent interest for psychological research, especially as Corollary 1 sets the foundation for research on more complex models. At first glance, the results appear to be in conceptional “conflict” with multi-level-models, since these often assume the random effects to be normally distributed and in this case there is no bounded interval, that includes the true parameter. However, even in these models there are fixed (hyper-)parameters one could know bounds for, and hence, it would be interesting for future research to analyze the behavior of these models in the external GMM framework. With respect to the limitation of robustness found in the simulation study, it would be interesting to investigate how robust the estimators are as a function of the length of the external interval. Finally, research on (the properties of) significance tests based on the use of an external intervals would be of great interest.

### Supplementary Information

Below is the link to the electronic supplementary material.

Supplementary file 1 (zip 5 KB)

Supplementary file 2 (pdf 318 KB)

## Declarations

### Data availability

The dataset of Pluck and Ruales-Chieruzzi (2021) analyzed during the current study is available in the PsychArchive repository, https://doi.org/10.23668/psycharchives.2897. All data generated by the simulations in this study are included in this article and its supplementary information files.
