# Supplementary material for: Using External Information for More Precise Inferences in General Regression Models
Source: Psychometrika. 2024 Feb 20;89(2):439–60. doi: 10.1007/s11336-024-09953-w (PMC11164816; doi:10.1007/s11336-024-09953-w)
Supplement: Supplementary file 2 — (pdf 318 KB) [file 11336_2024_9953_MOESM2_ESM.pdf]

# Using External Information for More Precise Inferences in General Regression Models: Supplementary Material II - Proofs and Tables

## 1 Introduction

This document presents the proofs of Corollary 1, Theorem 1, and the expressions in Table 1 and Table 2 in Section 3, as well as the results of the simulation study and the application of the externally informed linear model discussed in Sections 5 and 6 of the main paper. Each heading includes the relevant section in the main paper that cites the results presented under that heading.

# 2 Proofs (Section 3)

## 2.1 Corollary 1 (Section 3.1)

We start with the proof of Corollary 1:

**Corollary.** Assume $\hat{\boldsymbol{\theta}}_M$ is the GMM-estimator based on the model estimating equations alone (ignoring the external moments), and that $\mathbf{m}(\mathbf{z}, \boldsymbol{\theta})$ and $\boldsymbol{\theta}$ have the same dimension. Using the prerequisite $\mathbf{g}(\mathbf{z}, \boldsymbol{\theta}) = [\mathbf{m}(\mathbf{z}, \boldsymbol{\theta})^T, \mathbf{h}(\mathbf{z})^T]^T$ it follows, that $\boldsymbol{\Omega}$ has the block form

$$\boldsymbol{\Omega} = \begin{pmatrix} E[\mathbf{m}(\mathbf{z}, \boldsymbol{\theta})\mathbf{m}(\mathbf{z}, \boldsymbol{\theta})^T] & E[\mathbf{m}(\mathbf{z}, \boldsymbol{\theta})\mathbf{h}(\mathbf{z})^T] \\ E[\mathbf{h}(\mathbf{z})\mathbf{m}(\mathbf{z}, \boldsymbol{\theta})^T] & E[\mathbf{h}(\mathbf{z})\mathbf{h}(\mathbf{z})^T] \end{pmatrix} = \begin{pmatrix} \boldsymbol{\Omega}_M & \boldsymbol{\Omega}_R^T \\ \boldsymbol{\Omega}_R & \boldsymbol{\Omega}_h \end{pmatrix}$$

and that

$$\mathrm{Var}(\hat{\boldsymbol{\theta}}_{ex}) = \mathrm{Var}(\hat{\boldsymbol{\theta}}_M) - \frac{1}{n}\{E[\nabla_{\boldsymbol{\theta}}\mathbf{m}(\mathbf{z}, \boldsymbol{\theta}_0)]^T\}^{-1}\boldsymbol{\Omega}_R^T\boldsymbol{\Omega}_h^{-1}\boldsymbol{\Omega}_R\{E[\nabla_{\boldsymbol{\theta}}\mathbf{m}(\mathbf{z}, \boldsymbol{\theta}_0)]\}^{-1} \tag{1}$$

*Proof.* The block form of $\boldsymbol{\Omega}$ follows directly. The variance is $\mathrm{Var}(\hat{\boldsymbol{\theta}}_{ex}) = \frac{1}{n}(\mathbf{G}^T\mathbf{W}\boldsymbol{G})^{-1}$. Because $\mathbf{h}(\mathbf{z})$ does not depend on $\boldsymbol{\theta}$, we have $E(\nabla_{\boldsymbol{\theta}}\mathbf{h}(\mathbf{z})) = \mathbf{0}$, leading to $\mathbf{G} = E(\nabla_{\boldsymbol{\theta}}\mathbf{m}(\mathbf{z}, \boldsymbol{\theta}_0)^T, \mathbf{0})^T$. Using this form of $\mathbf{G}$ and partitioning $\mathbf{W}$ in the same way as $\boldsymbol{\Omega}$ leads to

$$\mathrm{Var}(\hat{\boldsymbol{\theta}}_{ex}) = \frac{1}{n}[E(\nabla_{\boldsymbol{\theta}}\mathbf{m}(\mathbf{z}, \boldsymbol{\theta}_0))^T]^{-1}\mathbf{W}_M^{-1}[E(\nabla_{\boldsymbol{\theta}}\mathbf{m}(\mathbf{z}, \boldsymbol{\theta}_0))]^{-1}$$

as $E(\nabla_{\boldsymbol{\theta}}\mathbf{m}(\mathbf{z}, \boldsymbol{\theta}_0))^T$ is a square matrix and is non-singular because both $\mathbf{W}_M$ and $\mathbf{G}^T\mathbf{W}\boldsymbol{G}$ are non-singular. Applying results for inverse blocks of partitioned matrices based on Schur complements (Chamberlain, 1987, p. 329, Lemma A.1.)

to $\mathbf{W}$ and $\mathbf{\Omega}$, leads to $\mathbf{W}_M^{-1} = \mathbf{\Omega}_M - \mathbf{\Omega}_R^T \mathbf{\Omega}_h^{-1} \mathbf{\Omega}_R$. This completes the proof, since $\mathrm{Var}(\hat{\boldsymbol{\theta}}_M) = \frac{1}{n}[E(\nabla_{\boldsymbol{\theta}} \mathbf{m}(\mathbf{z}, \boldsymbol{\theta}_0))^T]^{-1} \mathbf{\Omega}_M [E(\nabla_{\boldsymbol{\theta}} \mathbf{m}(\mathbf{z}, \boldsymbol{\theta}_0))]^{-1}$. □

## 2.2 Theorem 1 (Section 3.2)

Now we continue with the proof of Theorem 1.

**Theorem.** Let $\mathbf{H} = [\mathbf{h}(\mathbf{x}_1, y_1), \ldots, \mathbf{h}(\mathbf{x}_n, y_n)]^T$ be the $(n \times q)$ random matrix containing the externally informed sample moment functions and $\mathbf{1}_n$ a $(n \times 1)$-vector of ones. Further let $\hat{\mathbf{\Omega}}_h$ and $\hat{\mathbf{\Omega}}_R$ be consistent estimators of the corresponding matrices in Corollary 1. Then the (consistent) externally informed OLS estimator is

$$\hat{\boldsymbol{\beta}}_{ex} = (\mathbf{X}^T \mathbf{X})^{-1} \mathbf{X}^T \mathbf{y} - (\mathbf{X}^T \mathbf{X})^{-1} \hat{\mathbf{\Omega}}_R^T \hat{\mathbf{\Omega}}_h^{-1} \mathbf{H}^T \mathbf{1}_n$$

and its variance is

$$\begin{aligned} \mathrm{Var}(\hat{\boldsymbol{\beta}}_{ex}) &= \mathrm{Var}(\hat{\boldsymbol{\beta}}) - \mathbf{D} \\ &= \frac{1}{n} \sigma^2 [E(\mathbf{x}\mathbf{x}^T)]^{-1} - \frac{1}{n} [E(\mathbf{x}\mathbf{x}^T)]^{-1} \mathbf{\Omega}_R^T \mathbf{\Omega}_h^{-1} \mathbf{\Omega}_R [E(\mathbf{x}\mathbf{x}^T)]^{-1}, \end{aligned}$$

where $\sigma^2$ is the variance of the error in the assumed linear model.

The variance of the estimator shown in Theorem 1 can be seen as a special case of the variance formula in Corollary 1 and it was also derived by Hellerstein and Imbens (1999), hence we will only derive $\hat{\boldsymbol{\beta}}_{ex}$ here:

*Proof.* Using the notation of Definition 2, the regularity conditions are fulfilled for the externally informed linear model. The first order conditions for the GMM-estimator are $\hat{\mathbf{G}}^T \hat{\mathbf{W}} [\frac{1}{n} \sum_{i=1}^n \mathbf{g}(\mathbf{z}_i, \boldsymbol{\theta})] = \mathbf{0}$ (Newey & McFadden, 1994)[p.

2145], where $\hat{\mathbf{G}}$ is a consistent estimator for $\mathbf{G}$. In the multiple linear case $\frac{1}{n}\sum_{i=1}^{n}\mathbf{g}(\mathbf{z}_i,\boldsymbol{\theta})$ becomes $\begin{pmatrix}\frac{1}{n}\mathbf{X}^T(\mathbf{y}-\mathbf{X}\boldsymbol{\beta})\\ \frac{1}{n}\mathbf{H}^T\mathbf{1}\end{pmatrix}$ and it's $\hat{\mathbf{G}}$ is $\frac{1}{n}(\mathbf{X}^T\mathbf{X},\mathbf{0})^T$. Partitioning $\hat{\mathbf{W}}=\hat{\boldsymbol{\Omega}}^{-1}$ in the same manner as $\boldsymbol{\Omega}$ and solving for $\boldsymbol{\beta}$ we get

$$
\begin{aligned}
\mathbf{0} = \hat{\mathbf{G}}^T\hat{\mathbf{W}}[\frac{1}{n}\sum_{i=1}^{n}\mathbf{g}(\mathbf{z}_i,\boldsymbol{\theta})] &= \frac{1}{n}(\mathbf{X}^T\mathbf{X},\mathbf{0})\hat{\mathbf{W}}\begin{pmatrix}\frac{1}{n}\mathbf{X}^T(\mathbf{y}-\mathbf{X}\boldsymbol{\beta})\\ \frac{1}{n}\mathbf{H}^T\mathbf{1}\end{pmatrix}\\
&= \mathbf{X}^T\mathbf{X}\begin{pmatrix}\hat{\mathbf{W}}_M & \hat{\mathbf{W}}_R^T\end{pmatrix}\begin{pmatrix}\mathbf{X}^T(\mathbf{y}-\mathbf{X}\boldsymbol{\beta})\\ \mathbf{H}^T\mathbf{1}\end{pmatrix} = \hat{\mathbf{W}}_M\mathbf{X}^T(\mathbf{y}-\mathbf{X}\boldsymbol{\beta})+\hat{\mathbf{W}}_R^T\mathbf{H}^T\mathbf{1}\\
&\Rightarrow \hat{\mathbf{W}}_M\mathbf{X}^T\mathbf{X}\boldsymbol{\beta} = \hat{\mathbf{W}}_M\mathbf{X}^T\mathbf{y}+\hat{\mathbf{W}}_R^T\mathbf{H}^T\mathbf{1} \qquad (\text{multiply by } \hat{\mathbf{W}}_M^{-1} \text{ and } (\mathbf{X}^T\mathbf{X})^{-1})\\
&\Rightarrow \hat{\boldsymbol{\beta}}_{ex} = (\mathbf{X}^T\mathbf{X})^{-1}\mathbf{X}^T\mathbf{y}+(\mathbf{X}^T\mathbf{X})^{-1}\hat{\mathbf{W}}_M^{-1}\hat{\mathbf{W}}_R^T\mathbf{H}^T\mathbf{1}.
\end{aligned}
$$

The second order derivative is $-\mathbf{X}^T\mathbf{X}\hat{\mathbf{W}}_M\mathbf{X}^T\mathbf{X}$, which is negative definite if $\mathbf{X}$ has full column rank, which proves that $\hat{\boldsymbol{\beta}}_{ex}$ is indeed the searched maximum according to Definition 2. The structure of $\hat{\mathbf{W}}$ as a partitioned inverse provides the equality $\hat{\mathbf{W}}_M^{-1}\hat{\mathbf{W}}_R^T = -\hat{\boldsymbol{\Omega}}_R^T\hat{\boldsymbol{\Omega}}_h^{-1}$. This completes the proof. □

## 2.3 Expressions in Table 1 (Section 3.2)

We continue with the proof for the expressions in Table 1:

Forms of $\boldsymbol{\Omega}_R^T$ for various single moments

| moments | $h(\mathbf{x}, y)$ | $\boldsymbol{\Omega}_R^T$ |
|---|---|---|
| $E(y)$ | $y - E(y)_{ex}$ | $\sigma^2 E(\mathbf{x})$ |
| $E(x_j y)$ | $x_j y - E(x_j y)_{ex}$ | $\sigma^2 E(x_j \cdot \mathbf{x})$ |
| $E(y^2)$ | $y^2 - E(y^2)_{ex}$ | $2\sigma^2 E(\mathbf{x}\mathbf{x}^T)\boldsymbol{\beta}_0$ |
| $\sigma_y^2$ | $[y - E(y)]^2 - (\sigma_y^2)_{ex}$ | $2\sigma^2[E(\mathbf{x}\mathbf{x}^T)\boldsymbol{\beta}_0 - E(y)E(\mathbf{x})]$ |
| $\sigma_{x_j,y}$ | $[y - E(y)][x_j - E(x_j)] - (\sigma_{x_j,y})_{ex}$ | $\sigma^2 \boldsymbol{\sigma}_{x\cdot,x_j}$ |
| $\rho_{x_j,y}$ | $\frac{[y-E(y)][x_j-E(x_j)]}{\sigma_{x_j}\sigma_y} - (\rho_{x_j,y})_{ex}$ | $\frac{\sigma^2}{\sigma_{x_j}\sigma_y}\boldsymbol{\sigma}_{x\cdot,x_j}$ |
| $\beta_{x_j,y}$ | $\frac{[y-E(y)][x_j-E(x_j)]}{\sigma_{x_j}^2} - (\beta_{x_j,y})_{ex}$ | $\frac{\sigma^2}{\sigma_{x_j}^2}\boldsymbol{\sigma}_{x\cdot,x_j}$ |

Note: The subscript $ex$ indicates externally determined values. In the last line, $\beta_{x_j,y}$ represents the expected value of the estimator of the slope from a simple linear regression model, which is identical to the true value of the slope only if $x_j$ is independent of the other explanatory variables.

*Proof.* We only have to prove the correctness of the third column (the one for $\boldsymbol{\Omega}_R^T$). First we note, that $\boldsymbol{\Omega}_R^T = E(\mathbf{x}(y - \mathbf{x}^T\boldsymbol{\beta}_0)h(\mathbf{x}, y)) = E(\mathbf{x}\epsilon h(\mathbf{x}, y))$. We can omit the exact values of the external moments, as they are constants and as $\epsilon$ has the expected value 0. For the first row we get

$$E(\mathbf{x}\epsilon y) = E(\mathbf{x}\epsilon^2 + \epsilon\mathbf{x}\mathbf{x}^T\boldsymbol{\beta}_0) = E(\mathbf{x}\epsilon^2) = \sigma^2 E(\mathbf{x})$$

by the Gauss-Markov-assumptions. The second row follows by the same argument

just with the additional factor $x_j$. For the second moment of $y$ it follows that

$$\begin{aligned} E(\mathbf{x}\epsilon y^2) = E(\mathbf{x}\epsilon(\epsilon + \mathbf{x}^T\boldsymbol{\beta}_0)^2) &= E(\mathbf{x}\epsilon^3) + 2E(\epsilon^2\mathbf{x}\mathbf{x}^T\boldsymbol{\beta}_0) + E(\epsilon\mathbf{x}(\mathbf{x}^T\boldsymbol{\beta}_0)^2) \\ &= E(\mathbf{x})E(\epsilon^3) + 2\sigma^2 E(\mathbf{x}\mathbf{x}^T)\boldsymbol{\beta}_0. \end{aligned}$$

If the errors are assumed to be at least symmetrically distributed, the first summand vanishes, leaving the term written in the third row in Table 1. For the next row, we rewrite $(y - E(y))^2$ as $y^2 - 2yE(y) + E(y)^2$ and use the linearity of the expected value. Then the $\boldsymbol{\Omega}_R^T$ of the fourth row is just the one in the fourth row minus $2E(y)$ times the one in the second row. This is $2\sigma^2 E(\mathbf{x}\mathbf{x}^T)\boldsymbol{\beta}_0 - 2\sigma^2 E(\mathbf{x})E(Y)$, which is written in the fourth row. The expression in the fifth row is derived in the same manner as we can write

$$\mathbf{x}\epsilon(x_j - E(x_j))(y - E(y)) = \mathbf{x}\epsilon x_j y - \mathbf{x}\epsilon x_j E(y) - \mathbf{x}\epsilon y E(x_j) + \mathbf{x}\epsilon E(x_j)E(y).$$

The expected value of the second and the fourth term is zero, while the first term is equal to $\boldsymbol{\Omega}_R^T$ for the moment $E(x_j y)$ and the third term is equal to $\boldsymbol{\Omega}_R^T$ for the moment $E(y)$ times $E(x_j)$. The result is $\sigma^2 E(\mathbf{x}x_j) - \sigma^2 E(\mathbf{x})E(x_j)$, which is the vector of the covariances written in the fifth row. The last two rows follow from the fifth row, treating $\sigma_{x_j}$ and $\sigma_y$ as constants. □

## 2.4 Expressions in Table 2 (Section 3.2)

Effects of various single moments in terms of variance reduction.

| moments | $\mathbf{D}$ | effect on |
|---|---|---|
| $E(y)$ | $\frac{\sigma^4}{n\omega_h}\mathbf{e}_1\mathbf{e}_1^T$ | only $\beta_1$ |
| $E(x_jy)$ | $\frac{\sigma^4}{n\omega_h}\mathbf{e}_j\mathbf{e}_j^T$ | only $\beta_j$ |
| $E(y^2)$ | $\frac{4\sigma^4}{n\omega_h}\boldsymbol{\beta}_0\boldsymbol{\beta}_0^T$ | all $\beta_j \neq 0$ |
| $\sigma_y^2$ | $\frac{4\sigma^4}{n\omega_h}[\boldsymbol{\beta}_0 - E(y)\mathbf{e}_1][\boldsymbol{\beta}_0 - E(y)\mathbf{e}_1]^T$ | all $\beta_j \neq 0$ and $\beta_1$ |
| $\sigma_{x_j,y}$ | $\frac{\sigma^4}{n\omega_h}\tilde{\mathbf{e}}_j\tilde{\mathbf{e}}_j^T$ | $\beta_j$ and $\beta_1$ |
| $\rho_{x_j,y}$ | $\frac{\sigma^4}{n\omega_h\sigma_y^2\sigma_{x_j}^2}\tilde{\mathbf{e}}_j\tilde{\mathbf{e}}_j^T$ | $\beta_j$ and $\beta_1$ |
| $\beta_{x_j,y}$ | $\frac{\sigma^4}{n\omega_h\sigma_{x_j}^4}\tilde{\mathbf{e}}_j\tilde{\mathbf{e}}_j^T$ | $\beta_j$ and $\beta_1$ |

Note: The expression $\mathbf{e}_j$ denotes the $(p \times 1)$-vector with 1 at the j-th position and zeros elsewhere. Further we set $\tilde{\mathbf{e}}_j := -E(x_j)\cdot\mathbf{e}_1 + \mathbf{e}_j$. In the last line, $\beta_{x_j,y}$ represents the expected value of the estimator of the slope from a simple linear regression model, which is identical to the true value of the slope only if $x_j$ is independent of the other explanatory variables.

*Proof.* To prove the results in Table 2 it is sufficient to use Theorem 1. As $\omega_h$ is single valued, it holds that $\mathbf{D} = \frac{1}{n\omega_h}[E(\mathbf{x}\mathbf{x}^T)]^{-1}\boldsymbol{\Omega}_R^T\boldsymbol{\Omega}_R[E(\mathbf{x}\mathbf{x}^T)]^{-1}$. To derive $[E(\mathbf{x}\mathbf{x}^T)]^{-1}\boldsymbol{\Omega}_R^T$ the expressions of $\boldsymbol{\Omega}_R^T$ in Table 1 are used. The main idea is to factorize $E(\mathbf{x}\mathbf{x}^T)$ out of $\boldsymbol{\Omega}_R^T$. As $E(\mathbf{x}\cdot x_j) = E(\mathbf{x}\mathbf{x}^T)\mathbf{e}_j$ using the notation of Table 2 and noting that $x_1 = 1$, we get the results for $[E(\mathbf{x}\mathbf{x}^T)]^{-1}\boldsymbol{\Omega}_R^T$ in Table 6.

Table 6: Expressions for $[E(\mathbf{x}\mathbf{x}^T)]^{-1}\boldsymbol{\Omega}_R^T$ depending on the moment used.

| moments | $E(y)$ | $E(x_jy)$ | $E(y^2)$ | $\sigma_y^2$ | $\sigma_{x_j,y}$ | $\rho_{x_j,y}$ | $\beta_{x_j,y}$ |
|---|---|---|---|---|---|---|---|
| $[E(\mathbf{x}\mathbf{x}^T)]^{-1}\boldsymbol{\Omega}_R^T$ | $\sigma^2\mathbf{e}_1$ | $\sigma^2\mathbf{e}_j$ | $2\sigma^2\boldsymbol{\beta}_0$ | $2\sigma^2[\boldsymbol{\beta}_0 - E(y)\mathbf{e}_1]$ | $\sigma^2\tilde{\mathbf{e}}_j$ | $\frac{\sigma^2}{\sigma_y\sigma_{x_j}}\tilde{\mathbf{e}}_j$ | $\frac{\sigma^2}{\sigma_{x_j}^2}\tilde{\mathbf{e}}_j$ |

This proves the results in Table 2. □

To illustrate how to determine $\omega_h$, the case $E(y^2)$ is treated. Using $\epsilon \sim N(0, \sigma^2)$ and the Gauss-Markov-assumptions, we get

$$
\begin{aligned}
\omega_h &= E\{[y^2 - E(y^2)]^2\} = E\{[\epsilon^2 + 2\epsilon \mathbf{x}^T \boldsymbol{\beta}_0 + (\mathbf{x}^T \boldsymbol{\beta}_0)^2 - E(y^2)]^2\} \\
&= E(\epsilon^4) + E[(2\epsilon \mathbf{x}^T \boldsymbol{\beta}_0)^2] + 2E\{\epsilon^2[(\mathbf{x}^T \boldsymbol{\beta}_0)^2 - E(y^2)]\} + E\{[(\mathbf{x}^T \boldsymbol{\beta}_0)^2 - E(y^2)]^2\} \\
&+ 2E\{2\epsilon \mathbf{x}^T \boldsymbol{\beta}_0[(\mathbf{x}^T \boldsymbol{\beta}_0)^2 - E(y^2)]\} + E(2\epsilon^3 \mathbf{x}^T \boldsymbol{\beta}_0) \\
&= 3\sigma^4 + 4\sigma^2 E[(\mathbf{x}^T \boldsymbol{\beta}_0)^2] + 2\sigma^2 E[(\mathbf{x}^T \boldsymbol{\beta}_0)^2 - E(y^2)] + E\{[(\mathbf{x}^T \boldsymbol{\beta}_0)^2 - E(y^2)]^2\}.
\end{aligned}
$$

# References

Chamberlain, G. (1987). Asymptotic efficiency in estimation with conditional moment restrictions. *Journal of Econometrics*, *34*(3), 305–334. https://doi.org/10.1016/0304-4076(87)90015-7

Hellerstein, J. K., & Imbens, G. W. (1999). Imposing moment restrictions from auxiliary data by weighting. *The Review of Economics and Statistics*, *81*(1), 1–14. http://www.jstor.org/stable/2646780

Newey, W. K., & McFadden, D. (1994). Chapter 36 large sample estimation and hypothesis testing. Elsevier. https://doi.org/10.1016/S1573-4412(05)80005-4

# 3 Detailed results of the simulations (Section 5)

## 3.1 Correctly specified external moments (Section 5.2.1)

Table 7: Results of the simulations with correctly specified external moments for sample size $n = 30$.

| moments | $\beta_j$ | $\bar{\hat{\beta}}_{ex}$ | $\mathrm{Var}(\hat{\beta}_{ex})$ | $\overline{\widehat{\mathrm{Var}}(\hat{\beta}_{ex})}$ | $\hat{\Delta}_j$ | Cov | $\mathrm{Cov}_I$ | $\lvert CI\rvert$ | $\lvert\bigcup CI\rvert$ |
|---|---|---|---|---|---|---|---|---|---|
| $E(x_2)$ | $\beta_1$ | 0.979 | 0.880 | 0.866 | 0.000 | 0.950 | 0.950 | 3.757 | 3.757 |
| =OLS | $\beta_2$ | 0.489 | 0.077 | 0.087 | 0.000 | 0.970 | 0.970 | 1.184 | 1.184 |
| | $\beta_3$ | 2.077 | 1.383 | 1.326 | 0.000 | 0.934 | 0.934 | 4.675 | 4.675 |
| $E(y)$ | $\beta_1$ | 0.978 | 0.618 | 0.646 | 0.263 | 0.948 | 0.974 | 3.230 | 3.677 |
| $E(x_2y)$ | $\beta_2$ | 0.514 | 0.063 | 0.068 | 0.211 | 0.966 | 0.976 | 1.051 | 1.145 |
| $\sigma_{x_2,y}$ | $\beta_1$ | 0.860 | 0.663 | 0.628 | 0.261 | 0.934 | 0.944 | 3.207 | 3.364 |
| | $\beta_2$ | 0.549 | 0.027 | 0.026 | 0.687 | 0.958 | 0.978 | 0.649 | 0.727 |
| $\rho_{x_2,y}$ | $\beta_1$ | 0.911 | 0.676 | 0.619 | 0.272 | 0.932 | 0.944 | 3.183 | 3.337 |
| | $\beta_2$ | 0.523 | 0.021 | 0.025 | 0.700 | 0.970 | 0.982 | 0.634 | 0.710 |
| $\beta_{x_2,y}$ | $\beta_1$ | 0.928 | 0.615 | 0.618 | 0.270 | 0.952 | 0.954 | 3.183 | 3.332 |
| | $\beta_2$ | 0.515 | 0.014 | 0.025 | 0.697 | 0.994 | 0.994 | 0.633 | 0.707 |
| $E(x_3y)$ | $\beta_3$ | 2.109 | 1.076 | 1.008 | 0.233 | 0.950 | 0.968 | 4.082 | 4.421 |
| $\sigma_{x_3,y}$ | $\beta_1$ | 0.994 | 0.678 | 0.703 | 0.188 | 0.952 | 0.956 | 3.379 | 3.518 |
| | $\beta_3$ | 2.066 | 0.211 | 0.267 | 0.798 | 0.994 | 0.998 | 2.030 | 2.374 |
| $\rho_{x_3,y}$ | $\beta_1$ | 1.001 | 0.729 | 0.700 | 0.192 | 0.938 | 0.948 | 3.373 | 3.512 |
| | $\beta_3$ | 2.033 | 0.256 | 0.260 | 0.803 | 0.970 | 0.986 | 2.007 | 2.348 |
| $\beta_{x_3,y}$ | $\beta_1$ | 0.983 | 0.677 | 0.702 | 0.190 | 0.948 | 0.956 | 3.377 | 3.519 |
| | $\beta_3$ | 2.063 | 0.177 | 0.258 | 0.803 | 0.994 | 0.998 | 2.005 | 2.352 |
| $E(y^2)$ | $\beta_1$ | 0.991 | 0.887 | 0.848 | 0.020 | 0.932 | 0.952 | 3.717 | 3.843 |
| | $\beta_2$ | 0.495 | 0.074 | 0.083 | 0.043 | 0.968 | 0.976 | 1.158 | 1.209 |
| | $\beta_3$ | 2.103 | 1.374 | 1.260 | 0.050 | 0.922 | 0.938 | 4.554 | 4.770 |
| $\sigma_y^2$ | $\beta_1$ | 0.885 | 0.745 | 0.731 | 0.165 | 0.934 | 0.948 | 3.437 | 3.699 |
| | $\beta_2$ | 0.514 | 0.072 | 0.076 | 0.141 | 0.928 | 0.960 | 1.100 | 1.171 |
| | $\beta_3$ | 2.189 | 1.332 | 1.127 | 0.164 | 0.892 | 0.914 | 4.280 | 4.587 |

Note. The expressions $\bar{\hat{\beta}}_{ex}$, $\mathrm{Var}(\hat{\beta}_{ex})$, $\overline{\widehat{\mathrm{Var}}(\hat{\beta}_{ex})}$, $\hat{\Delta}_j$,$\lvert CI\rvert$ and $\lvert\bigcup CI\rvert$ are defined in the beginning of Section 5.2. The results for the moment $E(x_2)$ are equivalent to the OLS results. Cov is the coverage for the external point value and $\mathrm{Cov}_I$ symbolizes the coverage for the confidence interval union based on the external interval. Only the affected coefficients are reported per moment. The true values are $\beta_1 = 1$, $\beta_2 = 0.5$ and $\beta_3 = 2$.

Table 8: Results of the simulations with correctly specified external moments for sample size $n = 50$.

| moments | $\beta_j$ | $\bar{\hat{\beta}}_{ex}$ | $\mathrm{Var}(\hat{\beta}_{ex})$ | $\overline{\widehat{\mathrm{Var}}(\hat{\beta}_{ex})}$ | $\hat{\Delta}_j$ | Cov | $\mathrm{Cov}_I$ | $\|CI\|$ | $\|\bigcup CI\|$ |
|---|---|---|---|---|---|---|---|---|---|
| $E(x_2)$ | $\beta_1$ | 1.026 | 0.463 | 0.509 | 0.000 | 0.956 | 0.956 | 2.846 | 2.846 |
| | $\beta_2$ | 0.491 | 0.050 | 0.049 | 0.000 | 0.936 | 0.936 | 0.886 | 0.886 |
| | $\beta_3$ | 2.013 | 0.881 | 0.799 | 0.000 | 0.942 | 0.942 | 3.574 | 3.574 |
| $E(y)$ | $\beta_1$ | 1.024 | 0.355 | 0.366 | 0.285 | 0.952 | 0.976 | 2.408 | 2.865 |
| $E(x_2y)$ | $\beta_2$ | 0.506 | 0.038 | 0.038 | 0.227 | 0.946 | 0.968 | 0.778 | 0.873 |
| $\sigma_{x_2,y}$ | $\beta_1$ | 0.954 | 0.326 | 0.367 | 0.272 | 0.966 | 0.982 | 2.419 | 2.574 |
| | $\beta_2$ | 0.528 | 0.015 | 0.014 | 0.714 | 0.960 | 0.982 | 0.466 | 0.543 |
| $\rho_{x_2,y}$ | $\beta_1$ | 0.975 | 0.332 | 0.365 | 0.277 | 0.964 | 0.978 | 2.411 | 2.564 |
| | $\beta_2$ | 0.517 | 0.012 | 0.014 | 0.720 | 0.962 | 0.980 | 0.461 | 0.536 |
| $\beta_{x_2,y}$ | $\beta_1$ | 0.987 | 0.296 | 0.364 | 0.276 | 0.976 | 0.988 | 2.410 | 2.561 |
| | $\beta_2$ | 0.511 | 0.008 | 0.013 | 0.719 | 0.994 | 0.996 | 0.460 | 0.534 |
| $E(x_3y)$ | $\beta_3$ | 2.071 | 0.649 | 0.594 | 0.252 | 0.948 | 0.968 | 3.085 | 3.437 |
| $\sigma_{x_3,y}$ | $\beta_1$ | 1.019 | 0.357 | 0.410 | 0.196 | 0.966 | 0.978 | 2.551 | 2.689 |
| | $\beta_3$ | 2.049 | 0.137 | 0.133 | 0.834 | 0.982 | 0.996 | 1.412 | 1.764 |
| $\rho_{x_3,y}$ | $\beta_1$ | 1.020 | 0.383 | 0.409 | 0.198 | 0.956 | 0.966 | 2.548 | 2.687 |
| | $\beta_3$ | 2.037 | 0.157 | 0.131 | 0.837 | 0.942 | 0.972 | 1.403 | 1.754 |
| $\beta_{x_3,y}$ | $\beta_1$ | 1.016 | 0.355 | 0.410 | 0.196 | 0.964 | 0.974 | 2.550 | 2.690 |
| | $\beta_3$ | 2.036 | 0.103 | 0.130 | 0.837 | 0.986 | 0.996 | 1.400 | 1.751 |
| $E(y^2)$ | $\beta_1$ | 1.035 | 0.469 | 0.497 | 0.025 | 0.946 | 0.960 | 2.810 | 2.933 |
| | $\beta_2$ | 0.491 | 0.048 | 0.047 | 0.052 | 0.938 | 0.950 | 0.863 | 0.917 |
| | $\beta_3$ | 2.013 | 0.847 | 0.757 | 0.052 | 0.942 | 0.954 | 3.478 | 3.696 |
| $\sigma_y^2$ | $\beta_1$ | 0.989 | 0.382 | 0.428 | 0.166 | 0.956 | 0.982 | 2.602 | 2.871 |
| | $\beta_2$ | 0.501 | 0.046 | 0.043 | 0.144 | 0.932 | 0.946 | 0.821 | 0.895 |
| | $\beta_3$ | 2.055 | 0.810 | 0.685 | 0.150 | 0.912 | 0.940 | 3.296 | 3.604 |

Note. The expressions $\bar{\hat{\beta}}_{ex}$, $\mathrm{Var}(\hat{\beta}_{ex})$, $\overline{\widehat{\mathrm{Var}}(\hat{\beta}_{ex})}$, $\hat{\Delta}_j$,$|CI|$ and $|\bigcup CI|$ are defined in the beginning of Section 5.2. The results for the moment $E(x_2)$ are equivalent to the OLS results. Cov is the coverage for the external point value and $\mathrm{Cov}_I$ symbolizes the coverage for the confidence interval union based on the external interval. Only the affected coefficients are reported per moment. The true values are $\beta_1 = 1$, $\beta_2 = 0.5$ and $\beta_3 = 2$.

Table 9: Results of the simulations with correctly specified external moments for sample size $n = 100$.

| moments | $\beta_j$ | $\bar{\hat{\beta}}_{ex}$ | $\mathrm{Var}(\hat{\beta}_{ex})$ | $\overline{\widehat{\mathrm{Var}}(\hat{\beta}_{ex})}$ | $\hat{\Delta}_j$ | Cov | $\mathrm{Cov}_I$ | $\|CI\|$ | $\|\bigcup CI\|$ |
|---|---|---|---|---|---|---|---|---|---|
| $E(x_2)$ | $\beta_1$ | 0.968 | 0.252 | 0.246 | 0.000 | 0.952 | 0.952 | 1.962 | 1.962 |
| | $\beta_2$ | 0.512 | 0.024 | 0.024 | 0.000 | 0.958 | 0.958 | 0.606 | 0.606 |
| | $\beta_3$ | 2.020 | 0.377 | 0.383 | 0.000 | 0.944 | 0.944 | 2.449 | 2.449 |
| $E(y)$ | $\beta_1$ | 0.966 | 0.180 | 0.175 | 0.294 | 0.944 | 0.984 | 1.650 | 2.108 |
| $E(x_2y)$ | $\beta_2$ | 0.515 | 0.018 | 0.018 | 0.235 | 0.952 | 0.982 | 0.530 | 0.623 |
| $\sigma_{x_2,y}$ | $\beta_1$ | 0.954 | 0.174 | 0.178 | 0.276 | 0.954 | 0.976 | 1.666 | 1.819 |
| | $\beta_2$ | 0.519 | 0.007 | 0.006 | 0.731 | 0.944 | 0.986 | 0.312 | 0.388 |
| $\rho_{x_2,y}$ | $\beta_1$ | 0.964 | 0.184 | 0.177 | 0.280 | 0.944 | 0.960 | 1.662 | 1.815 |
| | $\beta_2$ | 0.513 | 0.006 | 0.006 | 0.735 | 0.966 | 0.996 | 0.309 | 0.385 |
| $\beta_{x_2,y}$ | $\beta_1$ | 0.972 | 0.164 | 0.177 | 0.279 | 0.960 | 0.978 | 1.662 | 1.813 |
| | $\beta_2$ | 0.509 | 0.003 | 0.006 | 0.734 | 0.994 | 0.996 | 0.309 | 0.384 |
| $E(x_3y)$ | $\beta_3$ | 2.030 | 0.273 | 0.281 | 0.262 | 0.962 | 0.990 | 2.101 | 2.459 |
| $\sigma_{x_3,y}$ | $\beta_1$ | 0.972 | 0.194 | 0.195 | 0.209 | 0.964 | 0.972 | 1.744 | 1.884 |
| | $\beta_3$ | 2.018 | 0.047 | 0.055 | 0.856 | 0.984 | 1.000 | 0.910 | 1.260 |
| $\rho_{x_3,y}$ | $\beta_1$ | 0.972 | 0.212 | 0.195 | 0.210 | 0.948 | 0.962 | 1.742 | 1.883 |
| | $\beta_3$ | 2.013 | 0.061 | 0.055 | 0.857 | 0.934 | 0.996 | 0.906 | 1.257 |
| $\beta_{x_3,y}$ | $\beta_1$ | 0.969 | 0.194 | 0.195 | 0.209 | 0.956 | 0.972 | 1.744 | 1.885 |
| | $\beta_3$ | 2.016 | 0.042 | 0.054 | 0.857 | 0.994 | 1.000 | 0.906 | 1.258 |
| $E(y^2)$ | $\beta_1$ | 0.973 | 0.260 | 0.241 | 0.022 | 0.938 | 0.958 | 1.940 | 2.054 |
| | $\beta_2$ | 0.512 | 0.022 | 0.022 | 0.059 | 0.962 | 0.974 | 0.588 | 0.645 |
| | $\beta_3$ | 2.020 | 0.348 | 0.361 | 0.055 | 0.942 | 0.960 | 2.379 | 2.604 |
| $\sigma_y^2$ | $\beta_1$ | 0.945 | 0.189 | 0.203 | 0.180 | 0.952 | 0.984 | 1.777 | 2.065 |
| | $\beta_2$ | 0.518 | 0.019 | 0.020 | 0.154 | 0.954 | 0.980 | 0.557 | 0.638 |
| | $\beta_3$ | 2.046 | 0.315 | 0.327 | 0.147 | 0.944 | 0.968 | 2.261 | 2.579 |

Note. The expressions $\bar{\hat{\beta}}_{ex}$, $\mathrm{Var}(\hat{\beta}_{ex})$, $\overline{\widehat{\mathrm{Var}}(\hat{\beta}_{ex})}$, $\hat{\Delta}_j$,$|CI|$ and $|\bigcup CI|$ are defined in the beginning of Section 5.2. The results for the moment $E(x_2)$ are equivalent to the OLS results. Cov is the coverage for the external point value and $\mathrm{Cov}_I$ symbolizes the coverage for the confidence interval union based on the external interval. Only the affected coefficients are reported per moment. The true values are $\beta_1 = 1$, $\beta_2 = 0.5$ and $\beta_3 = 2$.

## 3.2 Misspecified external moments (5.2.2)

Table 10: Results of the simulations with misspecified external moments for sample size $n = 15$.

| moments | $\beta_j$ | $\bar{\hat{\beta}}_{ex}$ | $\text{Var}(\hat{\beta}_{ex})$ | $\widehat{\text{Var}(\hat{\beta}_{ex})}$ | Cov | $\text{Cov}_I$ | \|CI\| | $\|\bigcup CI\|$ |
|---|---|---|---|---|---|---|---|---|
| $E(x_2)$ | $\beta_1$ | 0.982 | 2.210 | 2.096 | 0.926 | 0.926 | 5.676 | 5.676 |
| | $\beta_2$ | 0.499 | 0.228 | 0.223 | 0.964 | 0.964 | 1.843 | 1.843 |
| | $\beta_3$ | 2.128 | 3.110 | 3.148 | 0.966 | 0.966 | 7.051 | 7.051 |
| $E(y)$ | $\beta_1$ | 1.438 | 1.962 | 1.690 | 0.890 | 0.954 | 5.102 | 6.484 |
| $E(x_2y)$ | $\beta_2$ | 0.634 | 0.183 | 0.190 | 0.952 | 0.970 | 1.707 | 1.973 |
| $\sigma_{x_2,y}$ | $\beta_1$ | 0.547 | 1.581 | 1.612 | 0.896 | 0.924 | 5.012 | 5.546 |
| | $\beta_2$ | 0.723 | 0.117 | 0.092 | 0.910 | 0.966 | 1.187 | 1.452 |
| $\rho_{x_2,y}$ | $\beta_1$ | 0.647 | 1.280 | 1.593 | 0.934 | 0.948 | 4.945 | 5.481 |
| | $\beta_2$ | 0.672 | 0.102 | 0.088 | 0.958 | 0.978 | 1.154 | 1.417 |
| $\beta_{x_2,y}$ | $\beta_1$ | 0.711 | 1.337 | 1.560 | 0.914 | 0.932 | 4.931 | 5.424 |
| | $\beta_2$ | 0.640 | 0.057 | 0.083 | 0.968 | 0.984 | 1.141 | 1.384 |
| $E(x_3y)$ | $\beta_3$ | 2.525 | 2.206 | 2.560 | 0.958 | 0.980 | 6.418 | 7.348 |
| $\sigma_{x_3,y}$ | $\beta_1$ | 0.794 | 1.711 | 1.821 | 0.922 | 0.936 | 5.279 | 5.751 |
| | $\beta_3$ | 2.655 | 0.764 | 1.044 | 0.966 | 0.996 | 3.955 | 5.105 |
| $\rho_{x_3,y}$ | $\beta_1$ | 0.796 | 1.546 | 1.819 | 0.940 | 0.952 | 5.256 | 5.739 |
| | $\beta_3$ | 2.616 | 1.400 | 1.032 | 0.926 | 0.980 | 3.897 | 5.065 |
| $\beta_{x_3,y}$ | $\beta_1$ | 0.771 | 1.712 | 1.815 | 0.918 | 0.936 | 5.268 | 5.759 |
| | $\beta_3$ | 2.648 | 0.734 | 1.003 | 0.948 | 0.998 | 3.893 | 5.067 |
| $E(y^2)$ | $\beta_1$ | 1.046 | 2.124 | 2.108 | 0.896 | 0.914 | 5.726 | 6.081 |
| | $\beta_2$ | 0.563 | 0.234 | 0.225 | 0.916 | 0.952 | 1.856 | 1.985 |
| | $\beta_3$ | 2.343 | 2.964 | 3.151 | 0.928 | 0.954 | 7.085 | 7.585 |
| $\sigma_y^2$ | $\beta_1$ | 0.503 | 3.109 | 1.883 | 0.754 | 0.832 | 5.343 | 5.963 |
| | $\beta_2$ | 0.636 | 0.280 | 0.204 | 0.804 | 0.876 | 1.746 | 1.932 |
| | $\beta_3$ | 2.638 | 3.537 | 2.828 | 0.812 | 0.896 | 6.615 | 7.358 |

Note. The expressions $\bar{\hat{\beta}}_{ex}$, $\text{Var}(\hat{\beta}_{ex})$, $\widehat{\text{Var}(\hat{\beta}_{ex})}$,$|CI|$ and $|\bigcup CI|$ are defined in the beginning of Section 5.2. The results for the moment $E(x_2)$ are equivalent to the OLS results. Cov is the coverage for the external point value and $\text{Cov}_I$ symbolizes the coverage for the confidence interval union based on the external interval. Only the affected coefficients are reported per moment. The true values are $\beta_1 = 1$, $\beta_2 = 0.5$ and $\beta_3 = 2$.

Table 11: Results of the simulations with misspecified external moments for sample size $n = 30$.

| moments | $\beta_j$ | $\bar{\hat{\beta}}_{ex}$ | $\text{Var}(\hat{\beta}_{ex})$ | $\overline{\widehat{\text{Var}}(\hat{\beta}_{ex})}$ | Cov | $\text{Cov}_I$ | \|CI\| | $\lvert\bigcup CI\rvert$ |
|---|---|---|---|---|---|---|---|---|
| $E(x_2)$ | $\beta_1$ | 1.009 | 0.871 | 0.853 | 0.920 | 0.920 | 3.586 | 3.586 |
| | $\beta_2$ | 0.496 | 0.081 | 0.086 | 0.948 | 0.948 | 1.132 | 1.132 |
| | $\beta_3$ | 1.984 | 1.386 | 1.341 | 0.950 | 0.950 | 4.486 | 4.486 |
| $E(y)$ | $\beta_1$ | 1.588 | 0.726 | 0.644 | 0.852 | 0.980 | 3.142 | 4.665 |
| $E(x_2y)$ | $\beta_2$ | 0.623 | 0.063 | 0.070 | 0.936 | 0.974 | 1.032 | 1.301 |
| $\sigma_{x_2,y}$ | $\beta_1$ | 0.689 | 0.634 | 0.636 | 0.890 | 0.932 | 3.126 | 3.643 |
| | $\beta_2$ | 0.656 | 0.034 | 0.029 | 0.894 | 0.972 | 0.671 | 0.924 |
| $\rho_{x_2,y}$ | $\beta_1$ | 0.751 | 0.487 | 0.632 | 0.930 | 0.958 | 3.098 | 3.612 |
| | $\beta_2$ | 0.626 | 0.037 | 0.028 | 0.928 | 0.992 | 0.657 | 0.909 |
| $\beta_{x_2,y}$ | $\beta_1$ | 0.764 | 0.564 | 0.624 | 0.912 | 0.942 | 3.094 | 3.595 |
| | $\beta_2$ | 0.619 | 0.015 | 0.027 | 0.970 | 0.996 | 0.650 | 0.896 |
| $E(x_3y)$ | $\beta_3$ | 2.411 | 0.994 | 1.041 | 0.944 | 0.984 | 4.003 | 5.013 |
| $\sigma_{x_3,y}$ | $\beta_1$ | 0.795 | 0.670 | 0.700 | 0.910 | 0.934 | 3.250 | 3.767 |
| | $\beta_3$ | 2.529 | 0.226 | 0.287 | 0.872 | 0.998 | 2.061 | 3.315 |
| $\rho_{x_3,y}$ | $\beta_1$ | 0.819 | 0.578 | 0.701 | 0.930 | 0.954 | 3.244 | 3.752 |
| | $\beta_3$ | 2.462 | 0.564 | 0.287 | 0.822 | 0.986 | 2.043 | 3.278 |
| $\beta_{x_3,y}$ | $\beta_1$ | 0.779 | 0.671 | 0.699 | 0.904 | 0.934 | 3.249 | 3.778 |
| | $\beta_3$ | 2.532 | 0.188 | 0.277 | 0.814 | 0.998 | 2.040 | 3.312 |
| $E(y^2)$ | $\beta_1$ | 1.127 | 0.951 | 0.866 | 0.888 | 0.914 | 3.635 | 3.962 |
| | $\beta_2$ | 0.569 | 0.097 | 0.086 | 0.906 | 0.938 | 1.137 | 1.273 |
| | $\beta_3$ | 2.287 | 1.524 | 1.344 | 0.900 | 0.938 | 4.505 | 5.031 |
| $\sigma_y^2$ | $\beta_1$ | 0.456 | 1.456 | 0.744 | 0.716 | 0.790 | 3.321 | 4.051 |
| | $\beta_2$ | 0.645 | 0.117 | 0.077 | 0.772 | 0.880 | 1.058 | 1.268 |
| | $\beta_3$ | 2.619 | 1.900 | 1.187 | 0.760 | 0.882 | 4.180 | 5.008 |

Note. The expressions $\bar{\hat{\beta}}_{ex}$, $\text{Var}(\hat{\beta}_{ex})$, $\overline{\widehat{\text{Var}}(\hat{\beta}_{ex})}$,$|CI|$ and $|\bigcup CI|$ are defined in the beginning of Section 5.2. The results for the moment $E(x_2)$ are equivalent to the OLS results. Cov is the coverage for the external point value and $\text{Cov}_I$ symbolizes the coverage for the confidence interval union based on the external interval. Only the affected coefficients are reported per moment. The true values are $\beta_1 = 1$, $\beta_2 = 0.5$ and $\beta_3 = 2$.

Table 12: Results of the simulations with misspecified external moments for sample size $n = 100$.

| moments | $\beta_j$ | $\bar{\hat{\beta}}_{ex}$ | $\text{Var}(\hat{\beta}_{ex})$ | $\overline{\widehat{\text{Var}}(\hat{\beta}_{ex})}$ | Cov | $\text{Cov}_I$ | \|CI\| | $\|\bigcup CI\|$ |
|---|---|---|---|---|---|---|---|---|
| $E(x_2)$ | $\beta_1$ | 1.012 | 0.257 | 0.252 | 0.928 | 0.928 | 1.959 | 1.959 |
| | $\beta_2$ | 0.497 | 0.023 | 0.024 | 0.952 | 0.952 | 0.605 | 0.605 |
| | $\beta_3$ | 2.004 | 0.388 | 0.392 | 0.956 | 0.956 | 2.446 | 2.446 |
| $E(y)$ | $\beta_1$ | 1.666 | 0.233 | 0.184 | 0.606 | 0.994 | 1.677 | 3.383 |
| $E(x_2y)$ | $\beta_2$ | 0.621 | 0.017 | 0.019 | 0.850 | 0.994 | 0.543 | 0.831 |
| $\sigma_{x_2,y}$ | $\beta_1$ | 0.767 | 0.175 | 0.187 | 0.900 | 0.960 | 1.692 | 2.205 |
| | $\beta_2$ | 0.619 | 0.008 | 0.007 | 0.726 | 0.990 | 0.339 | 0.590 |
| $\rho_{x_2,y}$ | $\beta_1$ | 0.772 | 0.136 | 0.187 | 0.924 | 0.980 | 1.690 | 2.205 |
| | $\beta_2$ | 0.616 | 0.010 | 0.008 | 0.744 | 0.988 | 0.339 | 0.591 |
| $\beta_{x_2,y}$ | $\beta_1$ | 0.787 | 0.157 | 0.186 | 0.914 | 0.966 | 1.686 | 2.194 |
| | $\beta_2$ | 0.609 | 0.003 | 0.007 | 0.844 | 1.000 | 0.335 | 0.584 |
| $E(x_3y)$ | $\beta_3$ | 2.470 | 0.252 | 0.301 | 0.882 | 0.986 | 2.150 | 3.265 |
| $\sigma_{x_3,y}$ | $\beta_1$ | 0.805 | 0.192 | 0.201 | 0.902 | 0.966 | 1.749 | 2.289 |
| | $\beta_3$ | 2.533 | 0.048 | 0.057 | 0.354 | 0.996 | 0.925 | 2.258 |
| $\rho_{x_3,y}$ | $\beta_1$ | 0.800 | 0.160 | 0.201 | 0.914 | 0.974 | 1.748 | 2.293 |
| | $\beta_3$ | 2.538 | 0.157 | 0.057 | 0.432 | 0.970 | 0.923 | 2.264 |
| $\beta_{x_3,y}$ | $\beta_1$ | 0.801 | 0.194 | 0.201 | 0.896 | 0.968 | 1.748 | 2.292 |
| | $\beta_3$ | 2.532 | 0.043 | 0.056 | 0.332 | 0.998 | 0.921 | 2.258 |
| $E(y^2)$ | $\beta_1$ | 1.141 | 0.294 | 0.253 | 0.908 | 0.954 | 1.970 | 2.334 |
| | $\beta_2$ | 0.567 | 0.030 | 0.024 | 0.864 | 0.944 | 0.600 | 0.765 |
| | $\beta_3$ | 2.286 | 0.486 | 0.384 | 0.878 | 0.954 | 2.424 | 3.090 |
| $\sigma_y^2$ | $\beta_1$ | 0.547 | 0.580 | 0.209 | 0.630 | 0.840 | 1.772 | 2.695 |
| | $\beta_2$ | 0.625 | 0.039 | 0.021 | 0.708 | 0.914 | 0.558 | 0.813 |
| | $\beta_3$ | 2.523 | 0.641 | 0.337 | 0.700 | 0.906 | 2.256 | 3.288 |

Note. The expressions $\bar{\hat{\beta}}_{ex}$, $\text{Var}(\hat{\beta}_{ex})$, $\overline{\widehat{\text{Var}}(\hat{\beta}_{ex})}$,$|CI|$ and $|\bigcup CI|$ are defined in the beginning of Section 5.2. The results for the moment $E(x_2)$ are equivalent to the OLS results. Cov is the coverage for the external point value and $\text{Cov}_I$ symbolizes the coverage for the confidence interval union based on the external interval. Only the affected coefficients are reported per moment. The true values are $\beta_1 = 1$, $\beta_2 = 0.5$ and $\beta_3 = 2$.

# 4 Results of the application of the externally informed model (Section 6)

Table 13: Results using $\rho_{x,y} \in [.4, .85]$ and $E(y) = 100$.

| | | Pluck & Ruales-Chieruzzi | | | externally informed estimates | | |
|---|---|---|---|---|---|---|---|
| j | test | $\hat{\beta}_j$ | $s(\hat{\beta}_j)$ | $CI_{0.95}$ | $[\underline{\hat{\beta}_j}, \overline{\hat{\beta}_j}]$ | $[\underline{s(\hat{\beta}_j)}, \overline{s(\hat{\beta}_j)}]$ | $\bigcup CI_{0.95}$ |
| 1 | SpanLex | 54.61 | 8.864 | [37.06, 72.15] | [37.41, 66.90] | [2.336, 2.663] | [32.06, 71.90] |
| | WAT | 62.81 | 4.701 | [53.51, 72.12] | [60.02, 68.25] | [3.587, 3.689] | [52.77, 75.65] |
| | SCIRT | 60.81 | 4.395 | [52.11, 69.51] | [59.01, 65.48] | [3.910, 3.990] | [51.14, 73.50] |
| 2 | SpanLex | 1.821 | 0.332 | [1.163, 2.480] | [1.334, 2.430] | [0.124, 0.132] | [1.070, 2.696] |
| | WAT | 2.083 | 0.240 | [1.607, 2.559] | [1.773, 2.186] | [0.190, 0.196] | [1.379, 2.568] |
| | SCIRT | 3.292 | 0.358 | [2.583, 4.001] | [2.882, 3.393] | [0.309, 0.317] | [2.246, 4.015] |

Note. Note: The third and fourth columns contain the recomputed results of in terms of Pluck & Ruales-Chieruzzi (2021) the OLS regression coefficients $\hat{\beta}_j$, where $\hat{\beta}_1$ is the intercept and $\hat{\beta}_2$ is the slope and the robust standard errors $s(\hat{\beta}_j)$ of the coefficients. The (robust) 95% confidence intervals $CI_{0.95}$ for the parameters were computed in addition. The estimator interval $[\underline{\hat{\beta}_j}, \overline{\hat{\beta}_j}]$, the standard error interval $[\underline{s(\hat{\beta}_j)}, \overline{s(\hat{\beta}_j)}]$ and the 95% confidence interval union $\bigcup CI_{0.95}$ are shown as results of the estimation of the externally informed model.
